# Remnant cholesterol and auditory outcomes in NHANES 1999–2016: associations with frequency-range hearing loss and tinnitus

**DOI:** 10.1186/s12944-026-02910-9

**Published:** 2026-03-12

**Authors:** Jiyuan Yin, Haohong Lai, Qin Li, Haidi Yang

**Affiliations:** Department of Otolaryngology, Sun Yat-sen Memorial Hospital, Sun Yat-sen University, 107 West of Yan Jiang Road, Guangzhou, Guangdong 510120 China

**Keywords:** Hearing Loss, Tinnitus, Cholesterol, VLDL, Hyperlipidemias, NHANES

## Abstract

**Background:**

Evidence on the lipid determinants of auditory outcomes remains inconsistent. Remnant cholesterol (RC), the cholesterol fraction contained in triglyceride-rich lipoproteins, captures atherogenic and inflammatory burdens beyond conventional fractions. This study assessed the associations between RC and hearing loss across frequency ranges and between RC and tinnitus in a population-representative U.S. cohort. Identifying scalable metabolic markers may help support earlier recognition and prevention efforts to reduce avoidable hearing-related disability.

**Methods:**

Participants aged 40 years and older with valid audiometric evaluations and fasting lipid profiles were identified from the 1999–2016 National Health and Nutrition Examination Survey (NHANES) dataset. RC was estimated as total cholesterol minus the sum of low-density lipoprotein cholesterol (LDL-C) and high-density lipoprotein cholesterol (HDL-C) and was evaluated across predetermined concentration ranges. The outcomes included low-, speech-, and high-frequency hearing loss defined by air-conduction thresholds and self-reported tinnitus. Weighted multivariable logistic regression models were applied, sequentially adjusting for demographic, socioeconomic, lifestyle, and clinical covariates, followed by mutual adjustment for other lipid components. Subgroup and interaction analyses evaluated effect modification, including noise exposure.

**Results:**

In fully adjusted survey-weighted logistic models, higher RC was associated with hearing loss (OR 2.43, 95% CI 1.71–3.47) and with tinnitus (OR 1.64, 95% CI 1.31–2.06); category analyses indicated a monotonic increase (*P* for trend < 0.001). Associations displayed a clear dose–response across RC categories and remained robust in mutually adjusted models, whereas the inverse associations for HDL-C attenuated and became nonsignificant once RC was included. Analyses by frequency range revealed the strongest associations for high-frequency hearing loss, intermediate associations for speech-frequency hearing loss, and the weakest associations for low-frequency hearing loss. Subgroup findings were broadly consistent across strata, and interaction testing indicated amplified RC–auditory associations among participants reporting noise exposure.

**Conclusions:**

RC was linked to adverse auditory outcomes, including hearing loss and tinnitus, with clear dose–response patterns that persisted after mutual lipid adjustment. As a routinely available lipid-derived measure, RC may support scalable risk stratification to help prioritize earlier hearing evaluation and preventive counseling; confirmation in longitudinal cohorts and trials is needed.

**Supplementary Information:**

The online version contains supplementary material available at 10.1186/s12944-026-02910-9.

## Background

 Worldwide, approximately 1.5 billion people are affected by hearing loss (HL), and the global total is forecast to rise to about 2.4 billion by 2050 [1]. It is the third highest cause of years lived with disability [2] and creates a considerable economic burden through reduced productivity and increased health care costs [3]. HL is also a leading risk factor for tinnitus, a condition characterized by the perception of sound without external stimuli [4], which affects an estimated 740 million individuals globally [5]. Tinnitus not only contributes to daily functional impairment but is also associated with emotional distress, sleep disturbance, and heightened risks of depression and anxiety, thereby imposing a significant health care and economic burden [6, 7]. Despite the growing public health burden, current management of HL and tinnitus is largely focused on symptomatic and rehabilitative approaches, such as hearing aids, cochlear implants, sound therapy, and counseling, and there is still no universally effective curative treatment—particularly for chronic tinnitus and age-related HL—so many individuals continue to experience substantial residual symptoms and functional impairment [8, 9]. In this context, there is a need to better understand upstream mechanisms and to identify modifiable risk factors that could inform preventive or disease-modifying strategies.

Emerging evidence indicates that HL is related to an increased prevalence of cardiovascular disease (CVD) [10]. These epidemiological links, together with data showing systemic inflammatory activation and cochlear microvascular changes in individuals with HL and tinnitus, suggest that these auditory disorders may share common pathophysiological pathways with CVD, including vascular dysfunction and chronic low-grade inflammation [11–13]. Among the cardiometabolic factors that contribute to these pathways, dyslipidemia—characterized by alterations in conventional lipid fractions such as total cholesterol (TC), low-density lipoprotein cholesterol (LDL-C), and high-density lipoprotein cholesterol (HDL-C)—has attracted increasing attention because of its potential involvement in auditory dysfunction [7, 14, 15]. Statins, the most widely prescribed lipid-lowering agents for the primary prevention of CVD [16], have also been investigated as potential otoprotective agents. Experimental models and several observational studies suggest that statin therapy may mitigate noise-induced, age-related, metabolic-related, and ototoxic drug–induced sensorineural hearing loss [17–21]. However, epidemiological evidence directly linking cholesterol levels to HL is limited, and clinical findings on statin effectiveness remain inconsistent [22]. Parallel evidence also suggests that dyslipidemia in individuals with tinnitus with atherogenic lipid profiles—higher TC levels, higher LDL-C levels, and an unfavorable non–HDL-C to HDL-C ratio—is associated with greater tinnitus incidence and symptom burden [23–25]. Findings for statin use and tinnitus remain inconsistent across populations and study designs, likely reflecting differences in how tinnitus is defined or measured, variation in statin dosage and duration of use, and the influence of underlying health conditions that affect both statin prescription and tinnitus risk [26, 27]. Collectively, these observations highlight the need to further clarify how lipid abnormalities are related to HL and tinnitus.

In addition to conventional lipid fractions, remnant cholesterol (RC), often referred to as the “forgotten lipid,” has recently attracted considerable research interest [28]. RC refers to the cholesterol fraction within triglyceride-rich lipoproteins, mainly intermediate-density lipoprotein (IDL) and very-low-density lipoprotein (VLDL) during fasting, and chylomicron remnants in the nonfasting state [29]. Higher RC concentrations have been consistently linked to greater cardiovascular risk, particularly in middle-aged and older adults, and have demonstrated stronger predictive value for adverse events compared with conventional lipid measures [30, 31]. Mechanistically, RC contributes to systemic inflammation and microvascular injury by promoting endothelial dysfunction, oxidative stress, and vascular inflammation [32, 33]. These processes may compromise the cochlear microcirculation, disrupt the blood–labyrinth barrier, and increase the vulnerability of sensory hair cells and auditory neurons, thereby providing a plausible biological link between elevated RC levels and the development of HL and tinnitus.

Given the shared pathophysiological pathways between the cardiovascular and auditory systems, clarifying the relationship between RC and hearing outcomes may have important clinical implications. Adult hearing assessment is not routinely implemented in many primary care settings, and audiometric evaluation is typically initiated only after noticeable symptoms arise, which may delay the detection of subclinical hearing impairment. Because RC can be readily calculated from routine lipid panels, it was hypothesized that higher RC levels are associated with greater odds of hearing loss and tinnitus; if confirmed, RC could serve as a simple, inexpensive tool to help clinicians identify individuals who may benefit from timely audiologic evaluation. To clarify this unresolved issue, National Health and Nutrition Examination Survey (NHANES) data were analyzed to investigate the associations between RC and both HL and tinnitus among U.S. adults. To the best of current knowledge, no previous population-based study has systematically evaluated the relationship between RC and auditory outcomes.

## Methods

### Study design and population

This cross-sectional study analyzed data from the National Health and Nutrition Examination Survey (NHANES; RRID: SCR_013201) spanning 1999 to 2016. NHANES is a nationwide survey designed to represent the noninstitutionalized U.S. civilian population using a complex, multistage probability sampling framework. The protocol was approved by the National Center for Health Statistics (NCHS) Research Ethics Review Board, and all participants provided written informed consent. Participants are selected through a combination of geographic area sampling, household screening, and oversampling of specific demographic groups. Data collection consists of a structured household interview followed by a standardized physical examination and laboratory assessments conducted in mobile examination centers. Each 2-year cycle represents an independent cross-sectional sample, and NHANES is therefore not a longitudinal cohort; individual participants are not followed across cycles.

Among 101,316 participants from the 1999–2016 cycles, the analysis was restricted to adults aged ≥ 40 years. This threshold was chosen because previous epidemiological studies of RC and CVD have focused primarily on middle-aged and older people, in whom lipid abnormalities and vascular consequences are most relevant [31, 34, 35]. Participants without complete lipid profile data (TC, LDL-C, HDL-C) were further excluded.

**Fig. 1:**
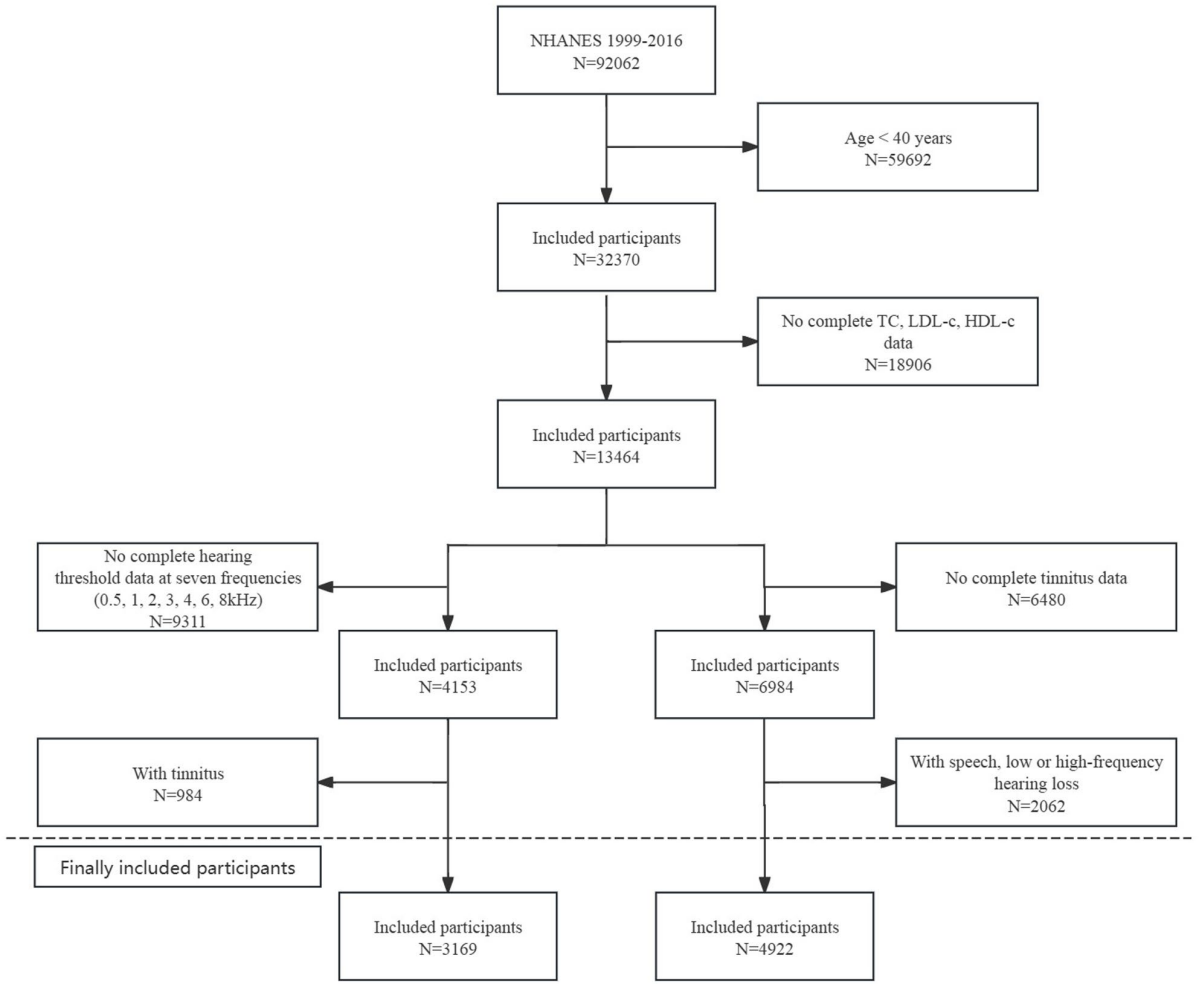
Flowchart of participant selection in NHANES 1999–2016. Flow diagram illustrating the selection of participants included in the analyses of hearing loss and tinnitus. Adults aged ≥ 40 years with complete audiometric assessments and fasting lipid data were included in the final analytic samples

Two analytic populations were then defined. For HL analysis, participants who reported tinnitus were excluded; thus, the hearing loss analytic sample included individuals with hearing loss but without self-reported tinnitus. For tinnitus analysis, participants with HL according to the audiometric definition described in the *Assessment of Auditory Outcomes* section were excluded so that this sample included individuals with tinnitus but without HL. To assess the robustness of this approach given the frequent co-occurrence of HL and tinnitus, a sensitivity analysis was also conducted in which participants with comorbid HL and tinnitus were retained and the coexisting outcome was controlled for in the fully adjusted models. A comprehensive flowchart illustrating how participants were selected is provided in Fig. 1.

### Assessment of auditory outcomes

Audiometric testing in NHANES was performed by trained examiners via standardized protocols in a sound-attenuated booth. Additional details regarding audiometric testing can be found in the NCHS Audiometry Procedures Manual. Pure-tone air conduction thresholds were measured at 0.5, 1, 2, 3, 4, 6, and 8 kHz for both ears. Pure-tone averages (PTA) were calculated for speech frequencies (SF-PTA: 0.5, 1, 2, and 4 kHz), low frequencies (LF-PTA: 0.5, 1, and 2 kHz), and high frequencies (HF-PTA: 3, 4, 6, and 8 kHz). In accordance with the latest WHO hearing loss grading framework [1], HL was defined as a PTA ≥ 20 dB in the better-hearing ear, consistent with the WHO/GBD-based revision of the normal-hearing threshold [36]. Accordingly, SFHL, LFHL, and HFHL were defined as SF-PTA ≥ 20 dB, LF-PTA ≥ 20 dB, and HF-PTA ≥ 20 dB, respectively. Overall, HL was defined as HL in any PTA-based frequency band (LFHL, SFHL, or HFHL), with each band defined as a PTA ≥ 20 dB in the better-hearing ear. Tinnitus status was determined based on participants’ responses to a standardized questionnaire item: *“During the past 12 months*,* have you been bothered by ringing*,* roaring*,* or buzzing in your ears or head lasting for five minutes or longer?”* Individuals who answered “yes” were categorized as having tinnitus.

### Lipid measurements and calculation of RC

During the NHANES physical examinations, venous blood was drawn after participants had fasted overnight and processed in certified laboratories using standardized analytic procedures. Enzymatic assays were employed to measure the serum concentrations of TC and triglycerides, whereas immunoassays were used to determine HDL-C levels. LDL-C was primarily calculated using the Friedewald equation, which derives LDL-C values from TC, TG, and HDL-C measurements.

RC was estimated indirectly as follows:

$$\:RC=TC-LDL\mathrm{-}C-HDL\mathrm{-}C$$

RC was examined as a continuous measure and in categories defined by cutoffs of < 0.50, 0.50–0.99, 1.00–1.49, and ≥ 1.50 mmol/L. This concentration-based classification has been commonly applied in recent studies, including investigations of RC and cardiovascular risk [37, 38].

### Covariates

Sociodemographic variables, including age, sex, race/ethnicity, educational attainment, marital status, and family poverty-income ratio (PIR), were collected through standardized NHANES interviews. Educational attainment was dichotomized as high school or less versus college or higher, while marital status was categorized as cohabiting (married or cohabiting) or alone (never married, widowed, divorced, or separated). The PIR was stratified into three levels: ≤130%, > 130–350%, and > 350%. Lifestyle factors, including smoking status, alcohol use, and noise exposure, were obtained from standardized NHANES questionnaires. Smoking status was ascertained from two standardized NHANES questions: *“Have you smoked at least 100 cigarettes in your entire life?”* and *“Do you now smoke cigarettes?”* Participants were grouped into never smokers (< 100 lifetime cigarettes), former smokers (≥ 100 lifetime cigarettes but not currently smoking), or current smokers (*≥* 100 lifetime cigarettes and currently smoking). Alcohol consumption was classified as nondrinking, moderate drinking (women: 0–15 g/day; men: 0–30 g/day), or heavy drinking (women: ≥15 g/day; men: ≥30 g/day). Environmental exposure included noise exposure, which was defined as any affirmative report of occupational, nonoccupational, or firearm-related noise [39]. Clinical conditions previously reported to be closely related to HL and tinnitus, including hypertension, diabetes, and CVD, were also considered [40–44]. Hypertension was identified using any of the following criteria: (1) self-reported physician diagnosis, (2) current antihypertensive medication use, or (3) a measured mean blood pressure of ≥ 140/90 mmHg during the examination. Diabetes was defined as (1) self-reported physician diagnosis, (2) use of insulin or oral hypoglycemic agents, (3) a fasting plasma glucose concentration ≥ 7.0 mmol/L, or (4) an HbA1c concentration ≥ 6.5%. CVD was defined based on self-reported physician diagnosis of congestive heart failure, coronary heart disease, angina, myocardial infarction, or stroke.

### Statistical analysis

Baseline characteristics were described using weighted means (with standard errors reported for continuous variables) and unweighted counts with weighted percentages for categorical variables. Comparisons between groups were performed using weighted *t*-tests for continuous variables, whereas Rao–Scott chi-square tests were used for categorical variables. Because NHANES uses a multistage, stratified cluster sampling design, all analyses were performed using survey-weighted methods to ensure the results were representative of the U.S. civilian, noninstitutionalized population. For each 2-year survey cycle, we applied the examination (subsample) weights provided by NHANES for participants with complete data on both exposure and outcome. When multiple cycles (1999–2016) were combined, multicycle weights were created by dividing the 2-year weights by the number of included cycles, in accordance with the NHANES analytic guidelines. In the survey design specification, the sample weights were included together with the masked variance strata and primary sampling units (PSUs), thereby accounting for unequal sampling probabilities, differential nonresponse, and poststratification. Associations between lipid parameters and auditory outcomes (overall HL, SFHL, LFHL, HFHL, and tinnitus) were examined via weighted multivariable logistic regression models. HL outcomes were analyzed as binary endpoints (presence vs. absence) using logistic regression, consistent with the threshold-based definition of HL. Three hierarchical survey-weighted logistic regression models were established. Model 1 accounted for age, sex, and race/ethnicity; Model 2 incorporated further adjustment for socioeconomic and lifestyle variables, including educational level, marital status, household PIR, smoking status, alcohol intake, and noise exposure; and Model 3 further accounted for clinical comorbidities such as hypertension, diabetes, and CVD. As a sensitivity analysis, the models were re-estimated without mutually excluding HL and tinnitus, and the fully adjusted model additionally included the coexisting outcome (tinnitus in HL models; HL in tinnitus models). To evaluate the independence of lipid parameters, supplementary models were fitted in which each lipid fraction was further adjusted for the remaining lipid measures. RC was examined both as a continuous variable and across predefined concentration categories (< 0.50, 0.50–0.99, 1.00–1.49, and ≥ 1.50 mmol/L). Linear trends were evaluated by assigning the median value of each RC category as a continuous term in regression models. Restricted cubic spline (RCS) models were additionally fitted to examine whether RC showed nonlinear relationships with HL (overall and frequency-specific) and tinnitus. In addition, subgroup analyses were performed to assess potential effect modification across demographic, lifestyle, and clinical factors. Multiple interaction terms were added to the regression models. In addition, dose‒response curves stratified by noise exposure were generated to visualize the RC‒auditory outcome relationships. The incorporation of survey weights, strata, and clusters allows appropriate variance estimation under this sampling structure. In NHANES, survey weights reflect the inverse probability of selection and incorporate adjustments for oversampling, survey nonresponse, and poststratification, ensuring that analyses yield nationally representative estimates. Strata refer to the masked variance strata constructed by the National Center for Health Statistics (NCHS) to support accurate variance estimation, whereas clusters correspond to PSUs representing sampled geographic areas. Incorporating survey weights, strata, and PSUs is therefore necessary to obtain unbiased population estimates and valid standard errors under a complex sampling design. All statistical analyses were carried out in R (v4.3.1; R Foundation for Statistical Computing, Vienna, Austria) via the *survey* package. Multivariate imputation via chained equations (MICE) was performed to handle missing covariate data, generating 100 complete datasets using the R package “mice”. Combined estimates across imputations were obtained via Rubin’s methodology [45, 46] to accommodate the complex survey design, yielding averaged point estimates and appropriately incorporating both within- and between-imputation variance components. Statistical significance was defined as a two-sided *P* < 0.05.

## Results

### Baseline characteristics of the study population

In total, 3,169 participants were evaluated for HL, and 4,922 were assessed for tinnitus. The weighted prevalence rates of HL and tinnitus were 44.9% and 22.4%, respectively. The participants with HL were older; more often male and non-Hispanic White; had lower educational attainment and PIRs; were more likely to be former smokers; reported noise exposure; and had hypertension, diabetes, and CVD. Participants with tinnitus were also older, more often non-Hispanic White, had lower education and income, had a higher prevalence of smoking, were more likely to report noise exposure, and more commonly had cardiometabolic conditions, although the sex distribution did not differ significantly.

Regarding lipid profiles, the HL and tinnitus groups exhibited elevated RC concentrations and reduced HDL-C levels. In addition, compared with participants without HL, participants with HL had decreased TC and LDL-C levels, whereas no significant differences in TC or LDL-C were observed between tinnitus and nontinnitus participants (Table 1).

**Table 1:** Weighted characteristics of the study population

Characteristics	Total	Non-HL	HL	*P* value	Total	Nontinnitus	Tinnitus	*P* value
Total	*n* = 3169	*n* = 1748	*n* = 1421		*n* = 4922	*n* = 3820	*n* = 1102	
Age, year	55.91 (0.36)	51.13 (0.29)	63.49 (0.48)	< 0.001	55.23 (0.26)	54.80 (0.28)	56.74 (0.52)	0.001
Sex, n (%)				< 0.001				0.233
Male	1502 (45.78)	664 (37.91)	838 (58.27)		2157 (41.44)	1684 (42.00)	473 (39.49)	
Female	1667 (54.22)	1084 (62.09)	583 (41.73)		2765 (58.56)	2136 (58.00)	629 (60.51)	
Race, n (%)				< 0.001				< 0.001
Mexican American	470 (5.13)	267 (5.59)	203 (4.41)		906 (5.42)	655 (5.42)	251 (5.43)	
Other Hispanic	278 (4.79)	165 (5.32)	113 (3.95)		330 (5.00)	285 (5.43)	45 (3.52)	
Non-Hispanic White	1397 (72.34)	629 (69.30)	768 (78.73)		2307 (72.52)	1692 (70.53)	615 (79.45)	
Non-Hispanic Black	722 (11.41)	494 (13.77)	228 (7.68)		1034 (11.38)	883 (12.47)	151 (7.60)	
Other Race	302 (6.33)	193 (7.02)	109 (5.23)		345 (5.68)	305 (6.15)	40 (4.00)	
BMI, kg/m²	29.22 (0.19)	29.26 (0.23)	29.13 (0.23)	0.639	28.84 (0.15)	28.87 (0.16)	28.73 (0.30)	0.642
Education level, n (%)				< 0.001				< 0.05
High school graduate or less	836 (15.79)	378 (13.05)	458 (20.11)		1512 (19.05)	1137 (18.14)	375 (22.23)	
College or above	2331 (84.21)	1369 (86.95)	962 (79.89)		3403 (80.95)	2677 (81.86)	726 (77.77)	
Marital status, n (%)				0.208				0.211
Cohabiting	2036 (71.27)	1129 (72.23)	907 (69.71)		3057 (69.62)	2391 (70.24)	666 (67.45)	
Alone	1101 (28.73)	596 (27.77)	505 (30.29)		1770 (30.38)	1364 (29.76)	406 (32.55)	
Family PIR, n (%)				< 0.001				0.001
< 1.3	752 (15.86)	393 (16.20)	359 (19.59)		1225 (17.65)	909 (16.90)	316 (20.20)	
1.3–3.5	1088 (33.98)	546 (30.17)	542 (40.12)		1701 (33.54)	1298 (32.52)	403 (37.03)	
≥ 3.5	1041 (50.16)	664 (53.63)	377 (40.29)		1584 (48.81)	1277 (50.58)	307 (42.77)	
Smoking, n (%)				< 0.001				< 0.001
Never a smoker	1663 (51.18)	1024 (57.08)	639 (41.77)		2542 (51.55)	2054 (53.68)	488 (44.13)	
Former smoker	952 (31.45)	405 (26.46)	547 (39.34)		1499 (30.38)	1109 (29.74)	390 (32.63)	
Current smoker	551 (17.37)	316 (16.46)	235 (18.89)		874 (18.07)	650 (16.58)	224 (23.24)	
Drinking, No. (%)				< 0.001				0.631
Nondrinker	489 (14.61)	255 (16.66)	234 (19.12)		719 (15.40)	558 (15.23)	161 (16.01)	
Moderate drinker	1051 (48.57)	572 (43.79)	479 (51.47)		1607 (48.77)	1257 (48.46)	350 (49.93)	
Heavy drinker	808 (36.82)	523 (39.55)	285 (29.41)		1148 (35.83)	914 (36.31)	234 (34.06)	
Noise exposure, n (%)				< 0.001				0.004
No	1725 (49.49)	1019 (53.69)	706 (42.83)		3169 (59.27)	2506 (60.61)	663 (54.59)	
Yes	1443 (50.51)	728 (46.31)	715 (57.17)		1751 (40.73)	1312 (39.39)	439 (45.41)	
Hypertension, n (%)				< 0.001				< 0.05
No	1762 (59.85)	1102 (65.93)	660 (50.17)		2857 (63.02)	2274 (63.99)	583 (59.61)	
Yes	1401 (40.15)	643 (34.07)	758 (49.83)		2044 (36.98)	1528 (36.01)	516 (40.39)	
Diabetes, n (%)				< 0.001				< 0.05
No	2591 (86.27)	1487 (89.26)	1104 (81.51)		4204 (88.82)	3275 (89.09)	929 (87.88)	
Yes	577 (13.73)	261 (10.74)	316 (18.49)		717 (11.12)	545 (10.91)	172 (12.12)	
CVD, n (%)				< 0.001				< 0.001
No	2768 (89.55)	1621 (94.24)	1147 (82.12)		4251 (89.96)	3348 (90.85)	903 (86.84)	
Yes	401 (10.45)	127 (5.76)	274 (17.88)		671 (10.04)	472 (9.15)	199 (13.16)	
TC, mmol/L	5.23 (0.03)	5.28 (0.04)	5.16 (0.04)	< 0.05	5.31 (0.02)	5.30 (0.03)	5.34 (0.04)	0.356
HDL-C, mmol/L	1.45 (0.01)	1.47 (0.02)	1.40 (0.02)	0.003	1.42 (0.01)	1.44 (0.01)	1.39 (0.02)	< 0.05
LDL-C, mmol/L	3.13 (0.03)	3.19 (0.03)	3.03 (0.04)	0.001	3.20 (0.02)	3.20 (0.02)	3.21 (0.04)	0.845
RC, mmol/L	0.66 (0.01)	0.62 (0.01)	0.72 (0.02)	< 0.001	0.68 (0.01)	0.67 (0.01)	0.74 (0.01)	< 0.001
RC, mmol/L, n (%)				< 0.001				< 0.001
< 0.50	1279 (40.47)	794 (45.61)	485 (32.31)		1757 (36.71)	1446 (38.79)	311 (29.46)	
0.50–0.99	1400 (43.88)	732 (41.58)	668 (47.52)		2309 (46.32)	1755 (45.39)	554 (49.58)	
1.00–1.49	381 (12.26)	167 (9.97)	214 (15.90)		655 (13.07)	473 (12.37)	182 (15.49)	
≥ 1.50	109 (3.39)	55 (2.84)	54 (4.27)		201 (3.90)	146 (3.45)	55 (5.47)	

Values are expressed as weighted means ± standard error for continuous variables and unweighted counts (weighted percentages) for categorical variables. Differences between groups were evaluated using weighted *t*-tests or Rao–Scott χ² tests

### Associations of lipid parameters with HL and tinnitus.

As shown in Table 2, RC was positively associated with both HL and tinnitus. According to the fully adjusted models (Model 3), RC was linked to increased odds of having HL (OR = 2.43, 95% CI: 1.71–3.47) and tinnitus (OR = 1.64, 95% CI: 1.31–2.06). RC was consistently associated with frequency-specific outcomes, including HFHL (OR = 2.21, 95% CI: 1.43–3.41), SFHL (OR = 1.77, 95% CI: 1.09–2.88), and LFHL (OR = 3.45, 95% CI: 1.20–9.91). In contrast, HDL-C was inversely associated with HL (OR = 0.69, 95% CI: 0.49–0.99) and tinnitus (OR = 0.72, 95% CI: 0.55–0.93), which was most evident for HFHL (OR = 0.60, 95% CI: 0.39–0.92). TC and LDL-C did not display consistent associations. To further evaluate the independence of these findings, each lipid parameter was additionally modeled with mutual adjustment for the other lipid fractions (Supplementary Table S1). RC remained positively associated with HL and tinnitus after adjustment for each of the other lipid parameters, whereas the inverse associations of HDL-C were attenuated and became nonsignificant once RC was included in the model (Model 6).

**Table 2:** Associations between cholesterol components and hearing loss and tinnitus

	Model 1		Model 2		Model 3	
	OR (95% CI)	*P* value	OR (95% CI)	*P* value	OR (95% CI)	*P* value
Hearing loss						
TC	1.019 (0.898, 1.155)	> 0.05	1.019 (0.897, 1.157)	> 0.05	1.032 (0.898, 1.187)	> 0.05
HDL-C	0.601 (0.418, 0.864)	0.007	0.681 (0.477, 0.973)	< 0.05	0.694 (0.486, 0.992)	< 0.05
LDL-C	0.984 (0.865, 1.119)	> 0.05	0.965 (0.846, 1.102)	> 0.05	0.977 (0.846, 1.129)	> 0.05
RC	2.698 (1.853, 3.929)	< 0.001	2.457 (1.726, 3.498)	< 0.001	2.434 (1.706, 3.473)	< 0.001
HFHL						
TC	1.000 (0.882, 1.133)	> 0.05	1.000 (0.881, 1.135)	> 0.05	1.013 (0.883, 1.164)	> 0.05
HDL-C	0.596 (0.412, 0.863)	0.007	0.673 (0.469, 0.966)	< 0.05	0.685 (0.477, 0.985)	< 0.05
LDL-C	0.966 (0.849, 1.099)	> 0.05	0.949 (0.831, 1.084)	> 0.05	0.961 (0.831, 1.111)	> 0.05
RC	2.632 (1.802, 3.846)	< 0.001	2.404 (1.682, 3.437)	< 0.001	2.383 (1.661, 3.418)	< 0.001
SFHL						
TC	0.936 (0.809, 1.083)	> 0.05	0.929 (0.800, 1.079)	> 0.05	0.917 (0.790, 1.064)	> 0.05
HDL-C	0.616 (0.400, 0.949)	< 0.05	0.673 (0.426, 1.063)	> 0.05	0.663 (0.416, 1.058)	> 0.05
LDL-C	0.921 (0.791, 1.073)	> 0.05	0.904 (0.771, 1.059)	> 0.05	0.888 (0.759, 1.039)	> 0.05
RC	1.989 (1.350, 2.931)	< 0.001	1.798 (1.200, 2.695)	0.004	1.822 (1.212, 2.737)	0.004
LFHL						
TC	1.045 (0.907, 1.202)	> 0.05	1.064 (0.924, 1.225)	> 0.05	1.059 (0.912, 1.229)	> 0.05
HDL-C	0.742 (0.478, 1.154)	> 0.05	0.903 (0.573, 1.422)	> 0.05	0.873 (0.556, 1.370)	> 0.05
LDL-C	1.018 (0.873, 1.187)	> 0.05	1.019 (0.870, 1.194)	> 0.05	1.010 (0.858, 1.190)	> 0.05
RC	2.096 (1.210, 3.630)	0.009	1.838 (1.023, 3.303)	< 0.05	1.889 (1.062, 3.361)	< 0.05
Tinnitus						
TC	1.014 (0.930, 1.104)	> 0.05	1.016 (0.933, 1.106)	> 0.05	1.027 (0.941, 1.120)	> 0.05
HDL-C	0.682 (0.520, 0.896)	0.006	0.708 (0.550, 0.913)	0.008	0.715 (0.553, 0.925)	< 0.05
LDL-C	1.007 (0.905, 1.122)	> 0.05	1.004 (0.903, 1.115)	> 0.05	1.017 (0.914, 1.131)	> 0.05
RC	1.704 (1.357, 2.141)	< 0.001	1.666 (1.334, 2.080)	< 0.001	1.642 (1.311, 2.056)	< 0.001

Weighted logistic regression models were sequentially adjusted: Model 1 for age, sex, and race; Model 2 additionally for socioeconomic and lifestyle factors (BMI, education, marital status, family PIR, smoking, drinking, and noise exposure); and Model 3 further for clinical comorbidities (hypertension, diabetes, and CVD). ORs and 95% CIs are presented for each lipid fraction

### RC categories and dose‒response analyses

When RC was divided into four categories (< 0.50, 0.50–0.99, 1.00–1.49, and ≥ 1.50 mmol/L), progressively higher levels were linked to a greater likelihood of HL and tinnitus. In the fully adjusted models, the odds of having HL were 1.57 (95% CI: 1.16–2.13), 2.33 (95% CI: 1.53–3.55), and 2.79 (95% CI: 1.55–5.04) across successive categories, whereas the corresponding odds of having tinnitus were 1.34 (95% CI: 1.10–1.63), 1.45 (95% CI: 1.11–1.90), and 1.95 (95% CI: 1.30–2.92) compared with the < 0.50 mmol/L group.

For frequency-specific outcomes, the strongest associations were observed for HFHL, where the odds increased to 1.56 (95% CI: 1.15–2.12), 2.21 (95% CI: 1.43–3.41), and 2.87 (95% CI: 1.58–5.19) across the 0.50–0.99, 1.00–1.49, and ≥ 1.50 mmol/L groups, respectively. Associations with SFHL were slightly weaker, with odds of 1.50 (95% CI: 1.04–2.15), 1.77 (95% CI: 1.09–2.88), and 2.41 (95% CI: 1.10–5.26), respectively. LFHL showed the weakest associations, with odds ratios (ORs) of 1.48 (95% CI: 0.99–2.21), 1.61 (95% CI: 0.96–2.69), and 3.45 (95% CI: 1.20–9.91), respectively.

**Fig. 2:**
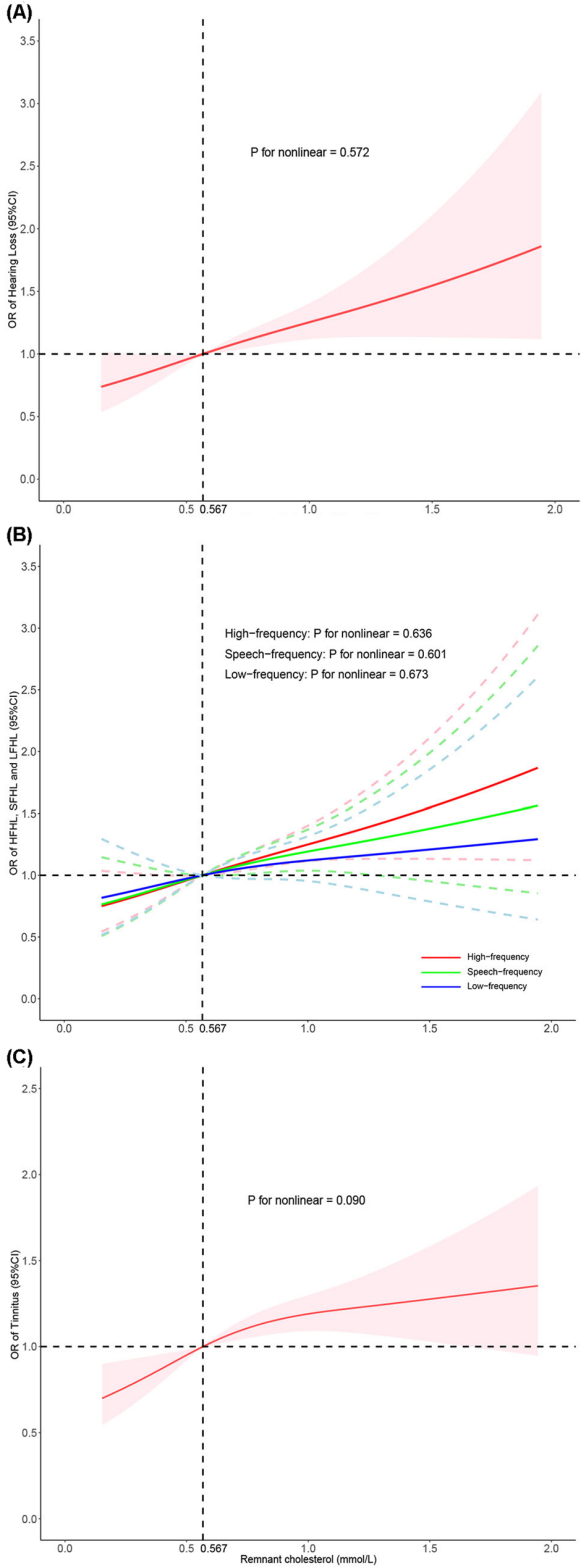
Restricted cubic spline analyses for the associations between remnant cholesterol and auditory outcomes. Restricted cubic spline models depicting the adjusted associations of RC with (**A**) overall hearing loss, (**B**) frequency-specific hearing loss (low-, speech-, and high-frequency), and (**C**) tinnitus. Analyses were weighted and adjusted for demographic, socioeconomic, lifestyle, and clinical covariates (Model 3). The solid lines represent adjusted odds ratios, and the shaded areas denote 95% CIs. In Panel B, the colored solid lines represent the adjusted odds ratios for each frequency range, and the two dotted lines of the same color denote the corresponding upper and lower bounds of the 95% CI for that frequency-range hearing loss. All panels share a common x-axis for the RC concentration (mmol/L)

Tests for linear trends were statistically significant for both overall HL and tinnitus, as were tests for HFHL, SFHL, and LFHL (all *P* values for trends < 0.05) (Table 3). RCS analyses further supported these findings, showing positive and approximately linear associations between RC and both HL and tinnitus, with frequency-specific analyses indicating the strongest relationships for HFHL, followed by SFHL, and the weakest for LFHL (Fig. 2). These results indicate that higher RC levels are associated with progressively greater odds of HL and tinnitus. The frequency-specific gradient suggests that high-frequency hearing thresholds have the strongest association with RC, followed by speech-frequency thresholds, whereas the association is weakest for low-frequency thresholds.

**Table 3:** Associations between remnant cholesterol levels and hearing loss and tinnitus

RC, mmol/L	Model 1		Model 2		Model 3	
	OR (95% CI)	*P* value	OR (95% CI)	*P* value	OR (95% CI)	*P* value
Hearing loss						
< 0.50	Reference		Reference		Reference	
0.50–0.99	1.615 (1.196, 2.181)	0.002	1.580 (1.169, 2.136)	0.003	1.574 (1.164, 2.127)	0.003
1.00–1.49	2.569 (1.666, 3.962)	< 0.001	2.341 (1.539, 3.562)	< 0.001	2.327 (1.526, 3.550)	< 0.001
≥ 1.50	3.277 (1.780, 6.034)	< 0.001	2.834 (1.567, 5.125)	< 0.001	2.793 (1.548, 5.039)	< 0.001
*P* for trend	<0.001		<0.001		<0.001	
HFHL						
<0.50	Reference		Reference		Reference	
0.50–0.99	1.600 (1.179, 2.173)	0.003	1.568 (1.154, 2.130)	0.004	1.562 (1.150, 2.122)	0.004
1.00–1.49	2.423 (1.547, 3.794)	<0.001	2.215 (1.435, 3.420)	<0.001	2.205 (1.425, 3.411)	<0.001
≥1.50	3.349 (1.820, 6.164)	<0.001	2.909 (1.603, 5.279)	<0.001	2.866 (1.583, 5.188)	<0.001
*P* for trend	＜0.001		＜0.001		＜0.001	
SFHL						
<0.50	Reference		Reference		Reference	
0.50–0.99	1.570 (1.111, 2.218)	<0.05	1.500 (1.037, 2.168)	<0.05	1.497 (1.040, 2.154)	<0.05
1.00–1.49	1.941 (1.203, 3.132)	0.007	1.770 (1.080, 2.902)	<0.05	1.772 (1.088, 2.884)	<0.05
≥1.50	2.783 (1.278, 6.062)	0.009	2.374 (1.092, 5.160)	<0.05	2.406 (1.100, 5.263)	<0.05
*P* for trend	＜0.001		0.003		0.003	
LFHL						
<0.50	Reference		Reference		Reference	
0.50–0.99	1.586 (1.078, 2.331)	<0.05	1.465 (0.996, 2.222)	>0.05	1.476 (0.987, 2.207)	>0.05
1.00–1.49	1.803 (1.074, 3.025)	<0.05	1.584 (0.940, 2.672)	>0.05	1.609 (0.964, 2.687)	>0.05
≥1.50	3.952 (1.390, 11.237)	<0.05	3.344 (1.160, 9.639)	<0.05	3.454 (1.204, 9.909)	<0.05
*P* for trend	0.003		<0.05		<0.05	
Tinnitus						
<0.50	Reference		Reference		Reference	
0.50–0.99	1.370 (1.120, 1.674)	0.003	1.343 (1.108, 1.629)	0.003	1.338 (1.099, 1.630)	0.004
1.00–1.49	1.525 (1.172, 1.985)	0.002	1.466 (1.123, 1.914)	0.005	1.453 (1.113, 1.897)	0.006
≥1.50	2.049 (1.341, 3.130)	<0.001	1.997 (1.345, 2.964)	<0.001	1.951 (1.302, 2.924)	0.001
*P* for trend	＜0.001		＜0.001		＜0.001	

Weighted logistic regression models were adjusted as shown in Table 2. RC was categorized into four concentration groups (<0.50, 0.50–0.99, 1.00–1.49, and ≥1.50 mmol/L).*P*-for-trend values were calculated by modeling the median RC value of each category as a continuous variable in the fully adjusted model

In sensitivity analyses retaining participants with comorbid HL and tinnitus and additionally adjusting for the coexisting outcome, the overall pattern of association remained similar, with higher RC categories continuing to show stronger associations, whereas the lowest RC category was not statistically significant after full adjustment (Supplementary Table S2).

## Subgroup and interaction analyses

**Fig. 3:**
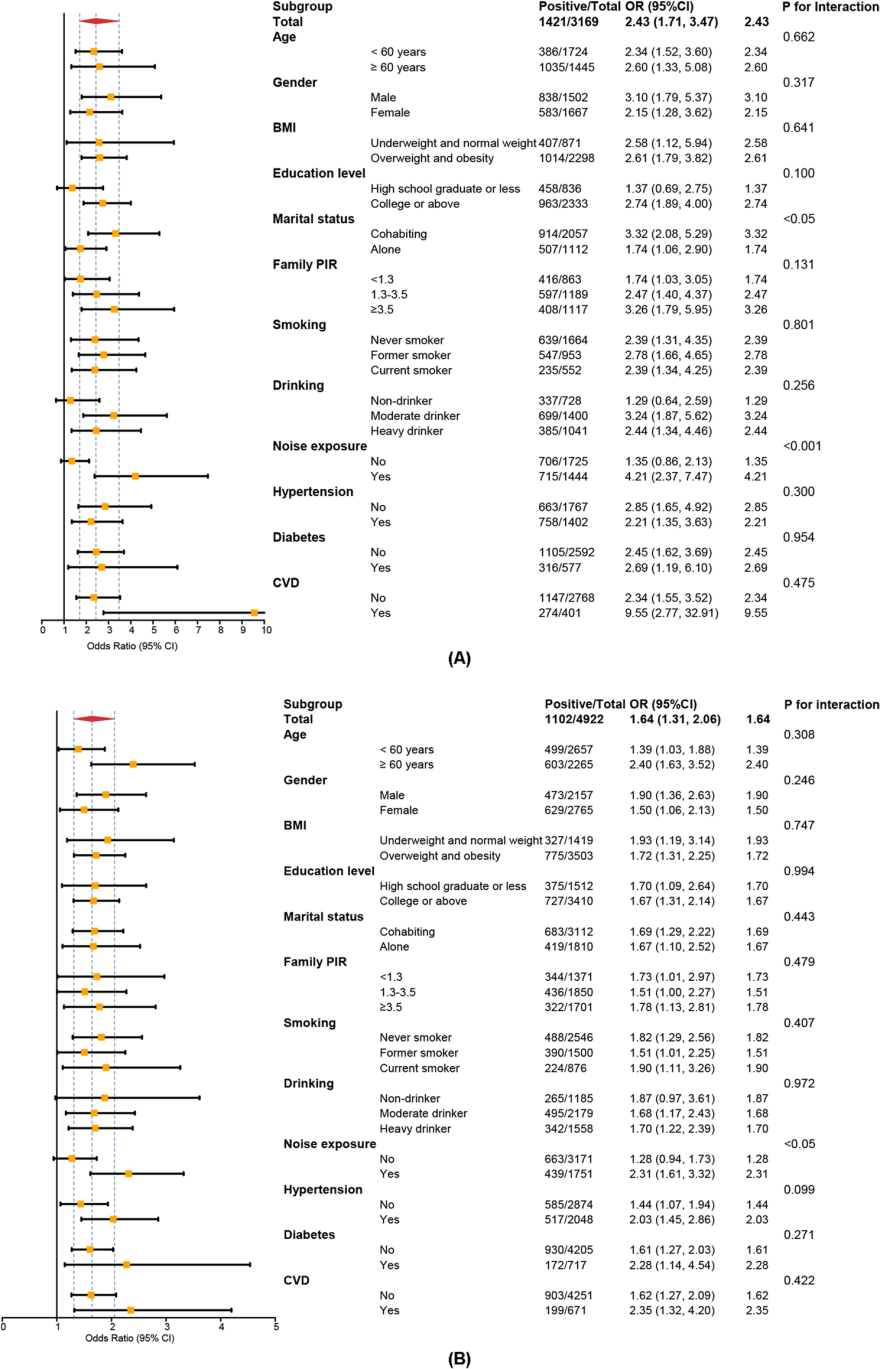
Subgroup analyses of the associations between remnant cholesterol and auditory outcomes. Forest plots showing subgroup-specific associations of remnant cholesterol with (**A**) hearing loss and (**B**) tinnitus. Weighted logistic regression models were adjusted as in Model 3. Points represent adjusted ORs, and horizontal bars indicate 95% CIs. P values for interactions were derived from cross-product terms between RC and each subgroup variable

Across various demographic, socioeconomic, lifestyle, and clinical subgroups, the subgroup analyses revealed that the relationships between RC and both HL and tinnitus remained largely consistent, reinforcing the robustness of the results (Fig. 3; detailed estimates in Supplementary Table S3). Significant associations were observed regardless of age, sex, Body mass index (BMI), smoking status, drinking status, marital status, education status, PIR, hypertension status, and diabetes status.

Interaction analyses, however, demonstrated heterogeneity due to noise exposure. For HL, the association with RC was significantly stronger among participants with self-reported noise exposure (OR = 4.21, 95% CI: 2.37–7.47; P for interaction < 0.001). Similarly, for tinnitus, stronger associations were also observed in noise-exposed individuals (OR = 2.31, 95% CI: 1.61–3.32; P for interaction < 0.05). RCS analyses stratified by noise exposure further confirmed this effect modification, showing steeper dose‒response relationships in exposed participants (Supplementary Figure S1). No significant interaction effects were observed for the remaining covariates.

## Discussion

In this population-based analysis of U.S. adults, higher RC levels were significantly associated with both HL and tinnitus. These associations were independent of demographic, socioeconomic, behavioral, and clinical factors and remained robust after mutual adjustment for other lipid fractions. Importantly, the strength of the association varied across frequency-specific outcomes, being most pronounced for HFHL, followed by SFHL, and weakest for LFHL. The associations were also consistent across most population subgroups, with stronger effects observed among individuals exposed to noise. To date, population-based evidence linking RC to auditory outcomes has been limited; this work offers the first population-based, comprehensive assessment of RC in relation to auditory outcomes and provides new evidence that RC may contribute to the development of auditory dysfunction. These associations were also materially unchanged in sensitivity analyses that retained participants with comorbid HL and tinnitus and controlled for the coexisting outcome, supporting the robustness of the main findings.

The evidence regarding conventional lipid fractions and auditory outcomes has been inconsistent. Although several studies have suggested that higher TC and LDL-C levels are associated with a greater likelihood of HL [10, 32], others have not confirmed these relationships [14, 47]. In this study, none of these conventional lipid parameters were significantly associated with HL or tinnitus. In contrast, higher HDL-C concentrations have been more consistently linked to a reduced risk of HL [14, 15, 47], although in the mutually adjusted models, the protective effect of HDL-C disappeared after adjustment for RC, suggesting that its association requires further confirmation. Taken together, these findings indicate that the relevance of traditional lipid fractions to auditory health may be limited, whereas RC provides a more specific marker of.

ic burden and vascular risk. Importantly, RC directly quantifies the cholesterol carried in triglyceride-rich remnants, thereby offering a more mechanistically grounded indicator of vascular and inflammatory stress. RC reflects the cholesterol content of triglyceride-rich remnants, thereby indicating the residual cholesterol burden that is mechanistically linked to systemic inflammation and microvascular dysfunction [33, 37, 38].

Several biological pathways may underlie the observed associations between RC and auditory outcomes. First, elevated RC promotes endothelial dysfunction and atherosclerosis, which can compromise the cochlear microcirculation and lead to ischemic injury to sensory hair cells [48–50]. Second, triglyceride-rich remnants are highly atherogenic and can trigger chronic inflammation and oxidative stress through macrophage activation, thereby exacerbating cochlear damage [20, 51]. Third, RC is strongly linked to metabolic dysregulation, including insulin resistance and type 2 diabetes, both of which have been linked to an increased likelihood of HL [52]. These multifactorial pathways should be viewed as potential explanations rather than confirmed mechanisms, and they may help illustrate why RC could relate more strongly to auditory dysfunction than conventional lipid measures, reinforcing their relevance as a potential clinical indicator.

Notably, the associations between RC and HL varied across frequency ranges. The effects were most pronounced for HFHL, followed by those for SFHL, whereas significant associations with LFHL were observed only at the highest RC levels. This pattern is biologically plausible, as the basal turn of the cochlea, which encodes high frequencies, is more vulnerable to vascular insufficiency and oxidative stress [51, 53]. Both mechanisms are strongly influenced by RC, which promotes endothelial dysfunction and chronic inflammation [49]. In addition, RC-mediated vascular injury may impair the blood–labyrinth barrier, further exacerbating cochlear ischemia and hair cell vulnerability, an area that deserves exploration in future mechanistic studies. Furthermore, interaction analyses revealed that the associations of RC with HL and tinnitus were amplified among individuals with noise exposure. Given that noise primarily damages outer hair cells in high-frequency regions [54], elevated RC levels may exacerbate cochlear injury through synergistic pathways involving microvascular dysfunction and inflammation. These results emphasize the importance of jointly considering metabolic and environmental risk factors when evaluating auditory impairment.

Importantly, statins—widely used for lowering lipids—reduce LDL-C and can also lower RC, although the degree of RC reduction is generally less pronounced than that of LDL-C [55] and may differ across statin types [56]. RC has been shown to predict cardiovascular risk beyond LDL-C and ApoB [35] and to be independently associated with incident cardiovascular events [34]. Previous clinical studies on statins and HL have yielded inconsistent results, with some reporting protective effects, particularly against drug-induced ototoxicity, while others reporting no significant benefit [17, 57]. Large cardiovascular trials have also demonstrated that even with maximal statin therapy or combination lipid-lowering regimens, considerable residual risk persists, and this residual risk is strongly related to RC [33, 37]. Similarly, residual risk may influence auditory outcomes, suggesting that targeted RC reduction strategies should be considered in future clinical studies on HL and tinnitus.

From a clinical and public health perspective, RC may serve as an easily obtainable indicator of an increased likelihood of HL and tinnitus. Because RC is already incorporated into routine lipid assessments for cardiovascular risk management, its use may provide complementary value for identifying individuals who warrant closer monitoring of hearing health. Notably, the associations between RC and auditory outcomes were stronger among individuals with noise exposure, underscoring the combined influence of metabolic and environmental stressors. However, this study neither establishes a specific RC threshold that should prompt referral for audiologic evaluation nor assesses whether incorporating RC improves the predictive performance beyond conventional demographic and clinical factors. Thus, RC should be interpreted as a preliminary risk indicator rather than a standalone decision-making tool. Given its simplicity and low cost, RC may nonetheless represent an efficient marker for population-level risk assessment in both general and occupational health contexts, and future work should determine clinically actionable cutoff values and evaluate its incremental predictive utility.

## Strengths and limitations

This study has several notable strengths, including a large, population-representative sample, standardized audiometric assessments, and comprehensive lipid profiling, which enhance both the validity and generalizability of the findings. In addition, the application of mutually adjusted models and subgroup analyses increases the robustness of the observed associations.

Several limitations should also be noted in considering these findings. First, the cross-sectional design precludes causal inference and does not establish temporal directionality between RC and auditory outcomes. Second, the analyses operationalized HL as a binary endpoint; therefore, associations with hearing loss severity (e.g., PTA as a continuous measure or graded severity categories) could not be evaluated. Third, tinnitus was assessed using a single self-report questionnaire item that referred to bothersome tinnitus during the past 12 months. This approach captures only the presence or absence of tinnitus and does not provide information on its laterality, severity, or functional impact, and reliance on a 12-month recall window may lead to misclassification, particularly among older adults. Moreover, RC was estimated via a calculation formula rather than direct measurement, which may increase the potential for measurement error. Overall, while these limitations warrant cautious interpretation, this study provides novel population-based evidence linking RC to HL and tinnitus and highlights the need for future prospective and mechanistic studies to clarify the underlying pathways involved.

## Conclusion

This study provides the first population-based evidence that elevated RC is independently associated with HL and tinnitus. These associations were particularly evident at higher frequencies and among individuals with noise exposure, underscoring the combined influence of metabolic and environmental risk factors. Because RC can be readily calculated from routine lipid panels, it may serve as a practical marker to help identify individuals who may benefit from earlier audiologic evaluation and reinforced hearing-conservation counseling, particularly where access to audiometry is limited, with the potential to reduce avoidable disability and preserve communication and quality of life. Prospective studies are needed to establish temporality and to evaluate whether RC-lowering strategies are associated with improved auditory outcomes, including HL severity.

## Supplementary Information

Supplementary Material 1.

Supplementary Material 2.

## Data Availability

The NHANES dataset (RRID: SCR\_013201) is publicly available at the National Center for Health Statistics of the Centers for Disease Control and Prevention (https://www.cdc.gov/nchs/nhanes/index.htm). The datasets used and/or analyzed during the current study are available from the corresponding author upon reasonable request.

## Abbreviations

BMI: Body mass index
CI: Confidence interval
CVD: Cardiovascular disease
dB HL: Decibels hearing level
HDL-C: High-density lipoprotein cholesterol
HFHL: High-frequency hearing loss
HF-PTA: High-frequency pure-tone average
HL: Hearing loss
IDL: Intermediate-density lipoprotein
LFHL: Low-frequency hearing loss
LF-PTA: Low-frequency pure-tone average
LDL-C: Low-density lipoprotein cholesterol
MICE: Multiple Imputation by Chained Equations
NHANES: National Health and Nutrition Examination Survey
OR: Odds ratio
PIR: Poverty-income ratio
PTA: Pure-tone average
PSUs: Primary sampling units
RC: Remnant cholesterol
RCS: Restricted cubic spline
SFHL: Speech-frequency hearing loss
SF-PTA: Speech-frequency pure-tone average
TC: Total cholesterol
VLDL: Very-low-density lipoprotein
