# Supplementary material for: Remnant cholesterol and auditory outcomes in NHANES 1999–2016: associations with frequency-range hearing loss and tinnitus
Source: Lipids Health Dis. 2026 Mar 12;25:114. doi: 10.1186/s12944-026-02910-9 (PMC13097959; doi:10.1186/s12944-026-02910-9)
Supplement: Supplementary file 1 — Supplementary Material 1. [file 12944_2026_2910_MOESM1_ESM.pdf]

Remnant cholesterol and auditory outcomes in NHANES 1999–2016: associations with frequency-range hearing loss and tinnitus

By jiyuan yin

WORD COUNT	4669	TIME SUBMITTED	30-DEC-2025 11:55PM
		PAPER ID	119717818

Remnant cholesterol and auditory outcomes in NHANES 1999–2016: associations with frequency-range hearing loss and tinnitus

Abstract

Background: Evidence on the lipid determinants of auditory outcomes remains inconsistent. Remnant cholesterol (RC), the cholesterol fraction contained in triglyceride-rich lipoproteins, captures atherogenic and inflammatory burdens beyond conventional fractions. This study assessed the associations between RC and hearing loss across frequency ranges, and between RC and tinnitus, in a population-representative U.S. cohort. Identifying scalable metabolic markers may help support earlier recognition and prevention efforts to reduce avoidable hearing-related disability.

Methods: Participants aged 40 years and older with valid audiometric evaluations and fasting lipid profiles were identified from the 1999–2016 National Health and Nutrition Examination Survey (NHANES) dataset. RC was estimated as total cholesterol minus the sum of low-density lipoprotein cholesterol (LDL-C) and high-density lipoprotein cholesterol (HDL-C), and was evaluated across predetermined concentration ranges. The outcomes included low-, speech-, and high-frequency hearing loss defined by air-conduction thresholds and self-reported tinnitus. Weighted multivariable logistic regression models were applied, sequentially adjusting for demographic, socioeconomic, lifestyle, and clinical covariates, followed by mutual adjustment for other lipid components. Subgroup and interaction analyses evaluated effect modification, including noise exposure.

Results: In fully adjusted survey-weighted logistic models, higher RC was associated with hearing loss (OR 2.43, 95% CI 1.71–3.47) and with tinnitus (OR 1.64, 95% CI 1.31–2.06); category analyses indicated a monotonic increase (*P* for trend < 0.001). Associations displayed a clear dose–response across RC categories and remained robust in mutually adjusted models, whereas the inverse associations for HDL-C attenuated and became nonsignificant once RC was included. Analyses by frequency range revealed the strongest associations for high-frequency hearing loss, intermediate associations for speech-frequency hearing loss, and the weakest associations for low-frequency hearing loss. Subgroup findings were broadly consistent across strata, and interaction testing indicated amplified RC–auditory associations among participants reporting noise exposure.

Conclusions: RC was linked to adverse auditory outcomes, including hearing loss and tinnitus, with clear dose–response patterns that persisted after mutual lipid adjustment. As a routinely available lipid-derived measure, RC may support scalable risk stratification to help prioritize earlier hearing evaluation and preventive counseling; confirmation in longitudinal cohorts and trials is needed.

Keywords: Hearing Loss; Tinnitus; Cholesterol, VLDL; Hyperlipidemias; NHANES

Background

Worldwide, approximately 1.5 billion people are affected by hearing loss (HL), and the global total is forecast to rise to about 2.4 billion by 2050 [1]. It is the third

highest cause of years lived with disability [2] and creates a considerable economic burden through reduced productivity and increased health care costs [3]. HL is also a leading risk factor for tinnitus, a condition characterized by the perception of sound without external stimuli [4], affecting an estimated 740 million individuals globally [5]. Tinnitus not only contributes to daily functional impairment but is also associated with emotional distress, sleep disturbance, and heightened risks of depression and anxiety, thereby imposing a significant health care and economic burden [6, 7]. Despite the growing public health burden, current management of HL and tinnitus is largely focused on symptomatic and rehabilitative approaches, such as hearing aids, cochlear implants, sound therapy, and counseling, and there is still no universally effective curative treatment—particularly for chronic tinnitus and age-related HL—so many individuals continue to experience substantial residual symptoms and functional impairment [8, 9]. In this context, there is a need to better understand upstream mechanisms and to identify modifiable risk factors that could inform preventive or disease-modifying strategies.

Emerging evidence indicates that HL is related to an increased prevalence of cardiovascular disease (CVD) [10]. These epidemiological links, together with data showing systemic inflammatory activation and cochlear microvascular changes in individuals with HL and tinnitus, suggest that these auditory disorders may share common pathophysiological pathways with CVD, including vascular dysfunction and chronic low-grade inflammation [11-13]. Among the cardiometabolic factors that contribute to these pathways, dyslipidemia—characterized by alterations in conventional lipid fractions such as total cholesterol (TC), low-density lipoprotein

cholesterol (LDL-C), and high-density lipoprotein cholesterol (HDL-C)—has attracted increasing attention because of its potential involvement in auditory dysfunction [7, 14, 15]. Statins, the most widely prescribed lipid-lowering agents for the primary prevention of CVD [16], have also been investigated as potential otoprotective agents. Experimental models and several observational studies suggest that statin therapy may mitigate noise-induced, age-related, metabolic-related, and ototoxic drug–induced sensorineural hearing loss [17-21]. However, epidemiological evidence directly linking cholesterol levels to HL is limited, and clinical findings on statin effectiveness remain inconsistent [22]. Parallel evidence also suggests that dyslipidemia in individuals with tinnitus, with atherogenic lipid profiles—higher TC levels, higher LDL-C levels, and an unfavorable non–HDL-C to HDL-C ratio—is associated with greater tinnitus incidence and symptom burden [23-25]. Findings for statin use and tinnitus remain inconsistent across populations and study designs, likely reflecting differences in how tinnitus is defined or measured, variation in statin dosage and duration of use, and the influence of underlying health conditions that affect both statin prescription and tinnitus risk [26, 27]. Collectively, these observations highlight the need to further clarify how lipid abnormalities are related to HL and tinnitus.

In addition to conventional lipid fractions, remnant cholesterol (RC), often referred to as the “forgotten lipid,” has recently attracted considerable research interest [28]. RC refers to the cholesterol fraction within triglyceride-rich lipoproteins, mainly intermediate-density lipoprotein (IDL) and very-low-density lipoprotein (VLDL) during fasting, and chylomicron remnants in the nonfasting state [29]. Higher RC

concentrations have been consistently linked to greater cardiovascular risk, particularly in middle-aged and older adults, and have demonstrated stronger predictive value for adverse events compared with conventional lipid measures [30, 31]. Mechanistically, RC contributes to systemic inflammation and microvascular injury by promoting endothelial dysfunction, oxidative stress, and vascular inflammation [32, 33]. These processes may compromise the cochlear microcirculation, disrupt the blood–labyrinth barrier, and increase the vulnerability of sensory hair cells and auditory neurons, thereby providing a plausible biological link between elevated RC levels and the development of HL and tinnitus.

Given the shared pathophysiological pathways between the cardiovascular and auditory systems, clarifying the relationship between RC and hearing outcomes may have important clinical implications. Adult hearing assessment is not routinely implemented in many primary care settings, and audiometric evaluation is typically initiated only after noticeable symptoms arise, which may delay the detection of subclinical hearing impairment. Because RC can be readily calculated from routine lipid panels, it was hypothesized that higher RC levels are associated with greater odds of hearing loss and tinnitus; if confirmed, RC could serve as a simple, inexpensive tool to help clinicians identify individuals who may benefit from timely audiologic evaluation. To clarify this unresolved issue, National Health and Nutrition Examination Survey (NHANES) data were analyzed to investigate the associations between RC and both HL and tinnitus among U.S. adults. To the best of current knowledge, no previous population-based study has systematically evaluated the relationship between RC and

auditory outcomes.

Methods

Study design and population

This cross-sectional study analyzed data from the National Health and Nutrition Examination Survey (NHANES; RRID:SCR_013201) spanning 1999 to 2016. NHANES is a nationwide survey designed to represent the noninstitutionalized U.S. civilian population using a complex, multistage probability sampling framework. The protocol was approved by the National Center for Health Statistics (NCHS) Research Ethics Review Board, and all participants provided written informed consent. Participants are selected through a combination of geographic area sampling, household screening, and oversampling of specific demographic groups. Data collection consists of a structured household interview followed by a standardized physical examination and laboratory assessments conducted in mobile examination centers. Each 2-year cycle represents an independent cross-sectional sample, and NHANES is therefore not a longitudinal cohort; individual participants are not followed across cycles.

Among 101,316 participants from the 1999–2016 cycles, the analysis was restricted to adults aged ≥40 years. This threshold was chosen because previous epidemiological studies of RC and CVD have focused primarily on middle-aged and older people, in whom lipid abnormalities and vascular consequences are most relevant [31, 34, 35]. Participants without complete lipid profile data (TC, LDL-C, HDL-C) were further excluded.

Two analytic populations were then defined. For HL analysis, participants who reported tinnitus were excluded; thus, the hearing loss analytic sample included individuals with hearing loss but without self-reported tinnitus. For tinnitus analysis, participants with HL according to the audiometric definition described in the *Assessment of Auditory Outcomes* section were excluded, so that this sample included individuals with tinnitus but without HL. Because HL and tinnitus commonly co-occur The NHANES tinnitus item does not indicate laterality; thus, unilateral and bilateral tinnitus cannot be distinguished. A comprehensive flowchart illustrating how participants were selected is provided in **Figure 1**.

Assessment of auditory outcomes

Audiometric testing in NHANES was performed by trained examiners via standardized protocols in a sound-attenuated booth. Additional details regarding audiometric testing can be found in the NCHS Audiometry Procedures Manual. Pure-tone air conduction thresholds were measured at 0.5, 1, 2, 3, 4, 6, and 8 kHz for both ears. Pure-tone averages (PTA) were calculated for speech frequencies (SF-PTA: 0.5, 1, 2, and 4 kHz), low frequencies (LF-PTA: 0.5, 1, and 2 kHz), and high frequencies (HF-PTA: 3, 4, 6, and 8 kHz). In accordance with the latest WHO hearing loss grading framework [1], HL was defined as a PTA ≥20 dB in the better-hearing ear, consistent with the WHO/GBD-based revision of the normal-hearing threshold [36]. Accordingly, SFHL, LFHL, and HFHL were defined as SF-PTA ≥20 dB, LF-PTA ≥20 dB, and HF-PTA ≥20 dB, respectively. Overall HL was defined as HL in any PTA-based frequency band

(LFHL, SFHL, or HFHL), with each band defined as a PTA ≥20 dB in the better-hearing ear. Tinnitus status was determined based on participants' responses to a standardized questionnaire item: *"During the past 12 months, have you been bothered by ringing, roaring, or buzzing in your ears or head lasting for five minutes or longer?"* Individuals who answered "yes" were categorized as having tinnitus.

Lipid measurements and calculation of RC

During the NHANES physical examinations, venous blood was drawn after participants had fasted overnight and processed in certified laboratories using standardized analytic procedures. Enzymatic assays were employed to measure the serum concentrations of TC and triglycerides, whereas immunoassays were used to determine HDL-C levels. LDL-C was primarily calculated using the Friedewald equation, which derives LDL-C values from TC, TG, and HDL-C measurements.

RC was estimated indirectly as follows:

$$RC = TC - LDL\text{-}C - HDL\text{-}C$$

RC was examined as a continuous measure and in categories defined by cutoffs of <0.50, 0.50–0.99, 1.00–1.49, and ≥1.50 mmol/L. This concentration-based classification has been commonly applied in recent studies, including investigations of RC and cardiovascular risk [37, 38].

Covariates

Sociodemographic variables, including age, sex, race/ethnicity, educational attainment, marital status, and family poverty-income ratio (PIR)—were collected through standardized NHANES interviews. Educational attainment was dichotomized as high school or less versus college or higher, while marital status was categorized as cohabiting (married or cohabiting) or alone (never married, widowed, divorced, or separated). The PIR was stratified into three levels: ≤130%, >130–350%, and >350%. Lifestyle factors, including smoking status, alcohol use, and noise exposure, were obtained from standardized NHANES questionnaires. Smoking status was ascertained from two standardized NHANES questions: *"Have you smoked at least 100 cigarettes in your entire life?"* and *"Do you now smoke cigarettes?"* Participants were grouped into never smokers (<100 lifetime cigarettes), former smokers (≥100 lifetime cigarettes but not currently smoking), or current smokers (≥ 100 lifetime cigarettes and currently smoking). Alcohol consumption was classified as nondrinking, moderate drinking (women: 0–15 g/day; men: 0–30 g/day), or heavy drinking (women: ≥15 g/day; men: ≥30 g/day). Environmental exposure included noise exposure, which was defined as any affirmative report of occupational, nonoccupational, or firearm-related noise [39]. Clinical conditions previously reported to be closely related to HL and tinnitus, including hypertension, diabetes, and CVD, were also considered [40-44]. Hypertension was identified using any of the following criteria: (1) self-reported physician diagnosis, (2) current antihypertensive medication use, or (3) a measured mean blood pressure of ≥140/90 mmHg during the examination. Diabetes was defined

as (1) self-reported physician diagnosis, (2) use of insulin or oral hypoglycemic agents, (3) a fasting plasma glucose concentration ≥7.0 mmol/L, or (4) an HbA1c concentration ≥6.5%. CVD was defined based on self-reported physician diagnosis of congestive heart failure, coronary heart disease, angina, myocardial infarction, or stroke.

Statistical analysis

Baseline characteristics were described using weighted means (with standard errors reported for continuous variables) and unweighted counts with weighted percentages for categorical variables. Comparisons between groups were performed using weighted *t* tests for continuous variables, whereas Rao–Scott chi-square tests were used for categorical variables. Because NHANES uses a multistage, stratified cluster sampling design, all analyses were performed using survey-weighted methods to ensure the results were representative of the U.S. civilian, noninstitutionalized population. For each 2-year survey cycle, we applied the examination (subsample) weights provided by NHANES for participants with complete data on both exposure and outcome. When multiple cycles (1999–2016) were combined, multicycle weights were created dividing the 2-year weights by the number of included cycles, in accordance with the NHANES analytic guidelines. In the survey design specification, the sample weights were included together with the masked variance strata and primary sampling units (PSUs), thereby accounting for unequal sampling probabilities, differential nonresponse, and poststratification. Associations between lipid parameters and auditory outcomes (overall HL, SFHL, LFHL, HFHL, and tinnitus) were examined

via weighted multivariable logistic regression models. HL outcomes were analyzed as binary endpoints (presence vs. absence) using logistic regression, consistent with the threshold-based definition of HL. Three hierarchical survey-weighted logistic regression models were established. Model 1 accounted for age, sex, and race/ethnicity; Model 2 incorporated further adjustment for socioeconomic and lifestyle variables, including educational level, marital status, and household PIR, smoking status, alcohol intake, and noise exposure; and Model 3 further accounted for clinical comorbidities such as hypertension, diabetes, and CVD. To evaluate the independence of lipid parameters, supplementary models were fitted in which each lipid fraction was further adjusted for the remaining lipid measures. RC was examined both as a continuous variable and across predefined concentration categories (<0.50, 0.50–0.99, 1.00–1.49, and ≥1.50 mmol/L). Linear trends were evaluated by assigning the median value of each RC category as a continuous term in regression models. Restricted cubic spline (RCS) analyses models were additionally fitted to examine whether RC showed nonlinear relationships with HL (overall and frequency-specific) and tinnitus. In addition, subgroup analyses were performed to assess potential effect modification across demographic, lifestyle, and clinical factors. Multiple interaction terms were add to the regression models. In addition, dose–response curves stratified by noise exposure were generated to visualize the RC–auditory outcome relationships. The incorporation of survey weights, strata, and clusters allows appropriate variance estimation under this sampling structure. In NHANES, survey weights reflect the inverse probability of selection and incorporate adjustments for oversampling, survey nonresponse, and

poststratification, ensuring that analyses yield nationally representative estimates. Strata refer to the masked variance strata constructed by the National Center for Health Statistics (NCHS) to support accurate variance estimation, whereas clusters correspond to PSUs representing sampled geographic areas. Incorporating survey weights, strata, and PSUs is therefore necessary to obtain unbiased population estimates and valid standard errors under a complex sampling design. All statistical analyses were carried out in R (v4.3.1; R Foundation for Statistical Computing, Vienna, Austria) via the *survey* package. Multivariate imputation via chained equations (MICE) was performed to handle missing covariate data, generating 100 complete using the R package "mice". Combined estimates across imputations were obtained via Rubin's methodology [45, 46] to accommodate the complex survey design, yielding averaged point estimates and appropriately incorporating both within- and between-imputation variance components. Statistical significance was defined as a two-sided $P < 0.05$.

Results

Baseline characteristics of the study population

In total, 3,169 participants were evaluated for HL, and 4,922 were assessed for tinnitus. The weighted prevalence rates of HL and tinnitus were 44.9% and 22.4%, respectively. The participants with HL were older; more often male and non-Hispanic White; had lower educational attainment and PIRs; were more likely to be former smokers; reported noise exposure; and had hypertension, diabetes, and CVD. The tinnitus participants were also older, more often non-Hispanic White, had lower

education and income, had a higher prevalence of smoking, were more likely to report noise exposure, and more commonly had cardiometabolic conditions, although the sex distribution did not significantly differ.

Regarding lipid profiles, the HL and tinnitus groups exhibited elevated RC concentrations and reduced HDL-C levels. In addition, compared with participants without HL, participants with HL had decreased TC and LDL-C levels, whereas no significant differences in TC or LDL-C were observed between tinnitus and nontinnitus participants (**Table 1**).

Associations of lipid parameters with HL and tinnitus

As shown in **Table 2**, RC was positively associated with both HL and tinnitus. According to the fully adjusted models (Model 3), RC was linked to increased odds of having HL (OR=2.43, 95% CI (Confidence interval): 1.71–3.47) and tinnitus (OR=1.64, 95% CI: 1.31–2.06). RC was consistently associated with frequency-specific outcomes, including HFHL (OR=2.21, 95% CI: 1.43–3.41), SFHL (OR=1.77, 95% CI: 1.09–2.88), and LFHL (OR=3.45, 95% CI: 1.20–9.91). In contrast, HDL-C was inversely associated with HL (OR=0.69, 95% CI: 0.49–0.99) and tinnitus (OR=0.72, 95% CI: 0.55–0.93), which was most evident for HFHL (OR=0.60, 95% CI: 0.39–0.92). TC and LDL-C did not display consistent associations. To further evaluate the independence of these findings, each lipid parameter was additionally modeled with mutual adjustment for the other lipid fractions (**Supplementary Table S1**). RC remained positively associated with HL and tinnitus after adjustment for each of the other lipid parameters, whereas

the inverse associations of HDL-C were attenuated and became nonsignificant once RC was included in the model (Model 6).

RC categories and dose–response analyses

When RC was divided into four categories (<0.50, 0.50–0.99, 1.00–1.49, and ≥1.50 mmol/L), progressively higher levels were linked to a greater likelihood of HL and tinnitus. In the fully adjusted models, the odds of having HL were 1.57 (95% CI: 1.16–2.13), 2.33 (95% CI: 1.53–3.55), and 2.79 (95% CI: 1.55–5.04) across successive categories, whereas the corresponding odds of having tinnitus were 1.34 (95% CI: 1.10–1.63), 1.45 (95% CI: 1.11–1.90), and 1.95 (95% CI: 1.30–2.92) compared with the <0.50 mmol/L group.

For frequency-specific outcomes, the strongest associations were observed for HFHL, where the odds increased to 1.56 (95% CI: 1.15–2.12), 2.21 (95% CI: 1.43–3.41), and 2.87 (95% CI: 1.58–5.19) across the 0.50–0.99, 1.00–1.49, and ≥1.50 mmol/L groups, respectively. Associations with SFHL were slightly weaker, with odds of 1.50 (95% CI: 1.04–2.15), 1.77 (95% CI: 1.09–2.88), and 2.41 (95% CI: 1.10–5.26) , respectively. LFHL showed the weakest associations, with odds ratios (OR) of 1.48 (95% CI: 0.99–2.21), 1.61 (95% CI: 0.96–2.69), and 3.45 (95% CI: 1.20–9.91), respectively.

Tests for linear trends were statistically significant for both overall HL and tinnitus, as were tests for HFHL, SFHL, and LFHL (all *P* values for trends <0.05) (**Table 3**). RCS analyses further supported these findings, showing positive and approximately linear associations between RC and both HL and tinnitus, with frequency-specific

analyses indicating the strongest relationships for HFHL, followed by SFHL, and the weakest for LFHL (**Figure 2**). These results indicate that higher RC levels are associated with progressively greater odds of HL and tinnitus. The frequency-specific gradient suggests that high-frequency hearing thresholds have the strongest association with RC, followed by speech-frequency thresholds, whereas the association is weakest for low-frequency thresholds.

Subgroup and interaction analyses

Across various demographic, socioeconomic, lifestyle, and clinical subgroups, the subgroup analyses revealed that the relationships between RC and both HL and tinnitus remained largely consistent, reinforcing the robustness of the results (**Figure 3**; detailed estimates in **Supplementary Table S2**). Significant associations were observed regardless of age, sex, Body mass index (BMI), smoking status and drinking status, marital status, education status, PIR, hypertension status, and diabetes status.

Interaction analyses, however, demonstrated heterogeneity due to noise exposure. For HL, the association with RC was significantly stronger among participants with self-reported noise exposure (OR=4.21, 95% CI: 2.37–7.47; P for interaction <0.001). Similarly, for tinnitus, stronger associations were also observed in noise-exposed individuals (OR=2.31, 95% CI: 1.61–3.32; P for interaction <0.05). RCS analyses stratified by noise exposure further confirmed this effect modification, showing steeper dose–response relationships in exposed participants (**Supplementary Figure S1**). No

significant interaction effects were observed for the remaining covariates.

Discussion

In this population-based analysis of U.S. adults, higher RC levels were significantly associated with both HL and tinnitus. These associations were independent of demographic, socioeconomic, behavioral, and clinical factors and remained robust after mutual adjustment for other lipid fractions. Importantly, the strength of the association varied across frequency-specific outcomes, being most pronounced for HFHL, followed by SFHL, and weakest for LFHL. The associations were also consistent across most population subgroups, with stronger effects observed among individuals exposed to noise. To date, population-based evidence linking RC to auditory outcomes has been limited; this work offers the first population-based, comprehensive assessment of RC in relation to auditory outcomes, offering new evidence that RC may contribute to the development of auditory dysfunction.

The evidence regarding conventional lipid fractions and auditory outcomes has been inconsistent. Although several studies have suggested that higher TC and LDL-C levels are associated with a greater likelihood of HL [10, 32], others have not confirmed these relationships [14, 47]. In this study, none of these conventional lipid parameters were significantly associated with HL or tinnitus. In contrast, higher HDL-C concentrations have been more consistently linked to a reduced risk of HL [14, 15, 47], although in the mutually adjusted models, the protective effect of HDL-C disappeared after adjustment for RC, suggesting that its association requires further confirmation.

Taken together, these findings indicate that the relevance of traditional lipid fractions to auditory health may be limited, whereas RC provides a more specific marker of atherogenic burden and vascular risk. Importantly, RC directly quantifies the cholesterol carried in triglyceride-rich remnants, thereby offering a more mechanistically grounded indicator of vascular and inflammatory stress. RC reflects the cholesterol content of triglyceride-rich remnants, thereby indicating the residual cholesterol burden that is mechanistically linked to systemic inflammation and microvascular dysfunction [33, 37, 38].

Several biological pathways may underlie the observed associations between RC and auditory outcomes. First, elevated RC promotes endothelial dysfunction and atherosclerosis, which can compromise the cochlear microcirculation and lead to ischemic injury to sensory hair cells [48-50]. Second, triglyceride-rich remnants are highly atherogenic and can trigger chronic inflammation and oxidative stress through macrophage activation, thereby exacerbating cochlear damage [20, 51]. Third, RC is strongly linked to metabolic dysregulation, including insulin resistance and type 2 diabetes, both of which have been linked to an increased likelihood of HL [52]. These multifactorial pathways should be viewed as potential explanations rather than confirmed mechanisms, and they may help illustrate why RC could relate more strongly to auditory dysfunction than conventional lipid measures, reinforcing their relevance as a potential clinical indicator.

Notably, the associations between RC and HL varied across frequency ranges. The effects were most pronounced for HFHL, followed by those for SFHL, whereas

significant associations with LFHL were observed only at the highest RC levels. This pattern is biologically plausible, as the basal turn of the cochlea, which encodes high frequencies, is more vulnerable to vascular insufficiency and oxidative stress [51, 53]. Both mechanisms are strongly influenced by RC, which promotes endothelial dysfunction and chronic inflammation [49]. In addition, RC-mediated vascular injury may impair the blood–labyrinth barrier, further exacerbating cochlear ischemia and hair cell vulnerability, an area that deserves exploration in future mechanistic studies. Furthermore, interaction analyses revealed that the associations of RC with HL and tinnitus were amplified among individuals with noise exposure. Given that noise primarily damages outer hair cells in high-frequency regions [54], elevated RCs may exacerbate cochlear injury through synergistic pathways involving microvascular dysfunction and inflammation. These results emphasize the importance of jointly considering metabolic and environmental risk factors when evaluating auditory impairment.

Importantly, statins—widely used for lowering lipids—reduce LDL-C and can also lower RC, although the degree of RC reduction is generally less pronounced than that of LDL-C [55] and may differ across statin types [56]. RC has been shown to predict cardiovascular risk beyond LDL-C and ApoB [35] and to be independently associated with incident cardiovascular events [34]. Previous clinical studies on statins and HL have yielded inconsistent results, with some reporting protective effects, particularly against drug-induced ototoxicity, while others reporting no significant benefit [17, 57]. Large cardiovascular trials have also demonstrated that even with

maximal statin therapy or combination lipid-lowering regimens, considerable residual risk persists, and this residual risk is strongly related to RC [33, 37]. Similarly, residual risk may influence auditory outcomes, suggesting that targeted RC reduction strategies should be considered in future clinical studies on HL and tinnitus.

From a clinical and public health perspective, RC may serve as an easily obtainable indicator of an increased likelihood of HL and tinnitus. Because RC is already incorporated into routine lipid assessments for cardiovascular risk management, its use may provide complementary value for identifying individuals who warrant closer monitoring of hearing health. Notably, the associations between RC and auditory outcomes were stronger among individuals with noise exposure, underscoring the combined influence of metabolic and environmental stressors. However, this study neither establishes a specific RC threshold that should prompt referral for audiologic evaluation, nor assesses whether incorporating RC improves the predictive performance beyond conventional demographic and clinical factors. Thus, RC should be interpreted as a preliminary risk indicator rather than a standalone decision-making tool. Given its simplicity and low cost, RC may nonetheless represent an efficient marker for population-level risk assessment in both general and occupational health contexts, and future work should determine clinically actionable cutoff values and evaluate its incremental predictive utility.

Strengths and limitations

This study has several notable strengths, including a large, population-

representative sample, standardized audiometric assessments, and comprehensive lipid profiling, which enhance both the validity and generalizability of the findings. In addition, the application of mutually adjusted models and subgroup analyses increases the robustness of the observed associations.

Several limitations should also be noted in considering these findings. Because HL and tinnitus commonly co-occur, excluding individuals with both conditions may limit generalizability and could introduce selection bias. Accordingly, the findings primarily characterize associations with isolated HL or isolated tinnitus rather than the comorbid phenotype. Because of the cross-sectional study design, causal inference is not possible, highlighting the need for longitudinal research to validate the temporal associations between RC and auditory outcomes. The analyses operationalized HL as a binary endpoint; therefore, associations with hearing loss severity (e.g., PTA as a continuous measure or graded severity categories) could not be evaluated. Tinnitus was assessed using a single self-report questionnaire item that referred to bothersome tinnitus during the past 12 months. This approach captures only the presence or absence of tinnitus and does not provide information on its laterality, severity, or functional impact, and reliance on a 12-month recall window may lead to misclassification, particularly among older adults. Moreover, RC was estimated via a calculation formula rather than direct measurement, which may increase the potential for measurement error. Overall, while these limitations warrant cautious interpretation, this study provides novel population-based evidence linking RC to HL and tinnitus and highlights the need for future prospective and mechanistic studies to clarify the underlying pathways involved.

Conclusion

This study provides the first population-based evidence that elevated RC is independently associated with HL and tinnitus. These associations were particularly evident at higher frequencies and among individuals with noise exposure, underscoring the combined influence of metabolic and environmental risk factors. Because RC can be readily calculated from routine lipid panels, it may serve as a practical marker to help identify individuals who may benefit from earlier audiologic evaluation and reinforced hearing-conservation counseling, particularly where access to audiometry is limited, with potential to reduce avoidable disability and preserve communication and quality of life. Prospective studies are needed to establish temporality and to evaluate whether RC-lowering strategies are associated with improved auditory outcomes, including HL severity.

Remnant cholesterol and auditory outcomes in NHANES 1999–2016: associations with frequency-range hearing loss and tinnitus

ORIGINALITY REPORT

15%
SIMILARITY INDEX

PRIMARY SOURCES

1	www.frontiersin.org<br>Internet	133 words — 2%
2	www.science.gov<br>Internet	51 words — 1%
3	www.mdpi.com<br>Internet	45 words — 1%
4	lipidworld.biomedcentral.com<br>Internet	43 words — 1%
5	public-pages-files-2025.frontiersin.org<br>Internet	43 words — 1%
6	pmc.ncbi.nlm.nih.gov<br>Internet	33 words — 1%
7	Emdin, Abby Louise. "Applying Machine Learning Methods to Describe Complex Medication Use in a Population of Community-Dwelling Older Adults Living With Dementia: Lessons for Pharmacoepidemiology Studies Using Health Administrative Data.", University of Toronto (Canada)<br>ProQuest	26 words — < 1%

8 Mahboubi, Hossein, Sepehr Oliaei, Saman Kiumehr, Sami Dwabe, and Hamid R. Djalilian. "The prevalence and characteristics of tinnitus in the youth population of the united states : Tinnitus in Youth Population", The Laryngoscope, 2013.
Crossref
26 words — < 1%

9 jamanetwork.com
Internet
26 words — < 1%

10 iris.unito.it
Internet
22 words — < 1%

11 academic.oup.com
Internet
21 words — < 1%

12 Huihui Sun, Jinzhi Yang, Li Ma, Yali wu. "Association between hs-CRP/HDL-C ratio and risk of prediabetes or diabetes: a cross-sectional study based on NHANES 2015–2023", BMC Endocrine Disorders, 2025
Crossref
19 words — < 1%

13 bmcpediatr.biomedcentral.com
Internet
19 words — < 1%

14 doaj.org
Internet
19 words — < 1%

15 Qixuan Wang, Xueling Wang, Lu Yang, Kun Han, Zhiwu Huang, Hao Wu. "Sex differences in noise-induced hearing loss: a cross-sectional study in China", Biology of Sex Differences, 2021
Crossref
17 words — < 1%

16 Zhifeng Chen, Yan Lu, Chenyu Chen, Shaolian Lin et al. "Association between tinnitus and hearing impairment among older adults with age-related hearing loss: a
17 words — < 1%

multi-center cross-sectional study", Frontiers in Neurology, 2024
Crossref

17 bmcgastroenterol.biomedcentral.com
Internet
17 words — < 1%

18 copsac.earlyvir.eu
Internet
16 words — < 1%

19 www.jlr.org
Internet
16 words — < 1%

20 inria.hal.science
Internet
15 words — < 1%

21 www.thieme-connect.de
Internet
13 words — < 1%

22 Enfa Zhao, Yiqing Chen, Hang Xie, Ruimeng Wang, Yuan Gao, Bingtian Dong, Chaoxue Zhang. "Association between the Endothelial Activation and Stress Index (EASIX) and all-cause and cardiovascular mortality in patients with diabetes and prediabetes", European Journal of Medical Research, 2025
Crossref
12 words — < 1%

23 www.cdc.gov
Internet
12 words — < 1%

24 Lin, Luotao. "Development, Comparison, and Interpretation of Temporal Lifestyle Behavior Patterns", Purdue University, 2025
ProQuest
11 words — < 1%

25 pure.johnshopkins.edu
Internet
11 words — < 1%

26 Giuseppe Mancia, Guido Grassi, Konstantinos P. Tsioufis, Anna F. Dominiczak, Enrico Agabiti Rosei. "Manual of Hypertension of the European Society of Hypertension", CRC Press, 2019 — 10 words — < 1%
Publications

27 Likang Li, Jun Lai, Jingyi Zhang, Harriette G C Van Spall, Lehana Thabane, Gregory Y H Lip, Guowei Li. "Remnant cholesterol and risk of premature mortality: An analysis from a nationwide prospective cohort study", European Heart Journal - Quality of Care and Clinical Outcomes, 2023 — 10 words — < 1%
Crossref

28 care.diabetesjournals.org — 10 words — < 1%
Internet

29 orthopedicreviews.openmedicalpublishing.org — 10 words — < 1%
Internet

30 Jiawei Peng, Jijun Wu, Xitu Luo, Chengyu Yang, Shian Wu, Wenjun Liu, Yuanhao Feng. "The cross-sectional association between cardiometabolic index and abdominal aortic calcification in U.S. adults: evidence from NHANES 2013–2014", Frontiers in Nutrition, 2025 — 9 words — < 1%
Crossref

31 Peng Jia, Rongwei Dong, Jiahao Li, Tongxu Wang et al. "Associations of the Hemoglobin-to-red blood cell distribution width ratio with Parkinson's Disease: the mediating role of systemic inflammation response index", Springer Science and Business Media LLC, 2025 — 9 words — < 1%
Crossref Posted Content

32 cardioprevent.ru — 9 words — < 1%
Internet

33	de Almeida Neves, Roseane Carvalho. "Inflammation-Related Risk Factors for Pancreatic Cancer: A Systematic Review with a Focus on Early Onset Cases", Universidade do Porto (Portugal), 2025<br>ProQuest	9 words — < 1%
34	journals.lww.com<br>Internet	9 words — < 1%
35	Mingxia Wu, Chunyu Hu, He Yu, Li Sun, Zongtao Chen. "Prevalence and Risk Factors of Plasma Adiponectin Deficiency: A Cross-Sectional Study in a Physical Examination Cohort from Southwest China", Diabetes, Metabolic Syndrome and Obesity, 2025<br>Crossref	8 words — < 1%
36	S A Mohiddin. "Novel association of hypertrophic cardiomyopathy, sensorineural deafness, and a mutation in unconventional myosin VI (MYO6)", Journal of Medical Genetics, 2004<br>Crossref	8 words — < 1%
37	pamw.pl<br>Internet	8 words — < 1%
38	Rui Du, Jie Liu, Xiaoyan Tang, Zili Chen, Lei Guan, WenHong Gao, Wei Huang. "Correlation of neutrophil-to-lymphocyte ratio and platelet-to-lymphocyte ratio with serum α-klotho levels in US middle-aged and older individuals: Results from NHANES 2007–2016", Preventive Medicine Reports, 2024<br>Crossref	6 words — < 1%
39	Yuchen Zhang, Yanqiang Zhang, Ziyue Fu, Chuanlu Shen, Kaile Wu, Yehai Liu. "Tinnitus and Risk of Mortality in Normal-Hearing U.S. Adults: A Weighted Study Adjusted for Covariate Balance", Life Conflux, 2025	6 words — < 1%

Crossref

EXCLUDE QUOTES	OFF	EXCLUDE SOURCES	OFF
EXCLUDE BIBLIOGRAPHY	OFF	EXCLUDE MATCHES	OFF
