# Supplementary material for: Remnant cholesterol and auditory outcomes in NHANES 1999–2016: associations with frequency-range hearing loss and tinnitus
Source: Lipids Health Dis. 2026 Mar 12;25:114. doi: 10.1186/s12944-026-02910-9 (PMC13097959; doi:10.1186/s12944-026-02910-9)

**Supplementary Table S1.** Associations between cholesterol components and auditory outcomes with mutual lipid adjustments。

|  | **Model 4** | | **Model 5** | | **Model 6** | |
| --- | --- | --- | --- | --- | --- | --- |
|  | **OR (95%CI)** | ***P* value** | **OR (95%CI)** | ***P* value** | **OR (95%CI)** | ***P* value** |
| **Hearing loss** | | | | | | |
| **TC** | 1.066 (0.932, 1.219) | >0.05 | 1.340 (0.949, 1.892) | >0.05 | 0.940 (0.817, 1.082) | >0.05 |
| **HDL-C** | 0.673 (0.474, 0.954) | <0.05 | 0.691 (0.482, 0.990) | <0.05 | 0.951 (0.658, 1.373) | >0.05 |
| **LDL-C** | 0.727 (0.510, 1.037) | >0.05 | 0.967 (0.835, 1.118) | >0.05 | 0.940 (0.820, 1.077) | >0.05 |
| **RC** | 2.559 (1.752, 3.739) | <0.001 | 2.376 (1.634, 3.455) | <0.001 | 2.478 (1.733, 3.543) | <0.001 |
|  | | | | | | |
| **HFHL** | | | | | | |
| **TC** | 1.047 (0.916, 1.197) | >0.05 | 1.305 (0.931, 1.831) | >0.05 | 0.924 (0.804, 1.062) | >0.05 |
| **HDL-C** | 0.670 (0.468, 0.958) | <0.05 | 0.681 (0.473, 0.980) | <0.05 | 0.929 (0.644, 1.340) | >0.05 |
| **LDL-C** | 0.734 (0.516, 1.043) | >0.05 | 0.950 (0.821, 1.099) | >0.05 | 0.924 (0.806, 1.060) | >0.05 |
| **RC** | 2.542 (1.728, 3.739) | <0.001 | 2.301 (1.584, 3.342) | <0.001 | 2.438 (1.695, 3.505) | <0.001 |
|  | | | | | | |
| **SFHL** | | | | | | |
| **TC** | 0.946 (0.816, 1.095) | >0.05 | 1.061 (0.724, 1.554) | >0.05 | 0.857 (0.735, 1.001) | >0.05 |
| **HDL-C** | 0.683 (0.426, 1.094) | >0.05 | 0.657 (0.412, 1.046) | >0.05 | 0.810 (0.494, 1.330) | >0.05 |
| **LDL-C** | 0.836 (0.560, 1.249) | >0.05 | 0.880 (0.751, 1.032) | >0.05 | 0.864 (0.739, 1.011) | >0.05 |
| **RC** | 2.043 (1.339, 3.118) | <0.001 | 1.641 (1.033, 2.607) | <0.05 | 1.889 (1.259, 2.833) | 0.002 |
|  | | | | | | |
| **LFHL** | | | | | | |
| **TC** | 1.075 (0.922, 1.254) | >0.05 | 1.340 (0.889, 2.022) | >0.05 | 1.005 (0.863, 1.170) | >0.05 |
| **HDL-C** | 0.838 (0.526, 1.336) | >0.05 | 0.873 (0.556, 1.371) | >0.05 | 1.128 (0.716, 1.777) | >0.05 |
| **LDL-C** | 0.751 (0.480, 1.175) | >0.05 | 1.007 (0.855, 1.187) | >0.05 | 0.986 (0.836, 1.162) | >0.05 |
| **RC** | 1.882 (1.044, 3.392) | <0.05 | 2.011 (1.066, 3.796) | <0.05 | 1.895 (1.063, 3.379) | <0.05 |
|  | | | | | | |
| **Tinnitus** | | | | | | |
| **TC** | 1.050 (0.961, 1.147) | >0.05 | 1.078 (0.895, 1.298) | >0.05 | 0.968 (0.875, 1.072) | >0.05 |
| **HDL-C** | 0.700 (0.540, 0.908) | 0.007 | 0.716 (0.552, 0.927) | <0.05 | 0.835 (0.647, 1.076) | >0.05 |
| **LDL-C** | 0.943 (0.746, 1.191) | >0.05 | 1.004 (0.901, 1.119) | >0.05 | 0.992 (0.888, 1.107) | >0.05 |
| **RC** | 1.690 (1.295, 2.205) | <0.001 | 1.523 (1.218, 1.905) | <0.001 | 1.647 (1.302, 2.082) | <0.001 |

Weighted logistic regression models were based on Model 3 (adjusted for demographic, socioeconomic, lifestyle, and clinical covariates). To assess the independence among lipid fractions, additional models (Models 4–6) were constructed by sequentially adjusting for other lipid components. Specifically, when total cholesterol (TC) was the exposure, Models 4–6 were further adjusted for HDL-C, LDL-C, and RC, respectively; when HDL-C was the exposure, Models 4–6 were adjusted for TC, LDL-C, and RC; when LDL-C was the exposure, Models 4–6 were adjusted for TC, HDL-C, and RC; and when RC was the exposure, Models 4–6 were adjusted for TC, HDL-C, and LDL-C. ORs (95% CIs) indicate the associations between each lipid parameter and auditory outcomes after these mutual adjustments.

**Supplementary Table S2.** Associations between remnant cholesterol levels and hearing loss (n=4153) and tinnitus (n=6984).

| RC, mmol/L | Model 1 | | Model 2 | | Model 3 | |
| --- | --- | --- | --- | --- | --- | --- |
|  | OR (95% CI) | *P* value | OR (95% CI) | *P* value | OR (95% CI) | *P* value |
| Hearing loss | | | | | | |
| <0.50 | Reference |  | Reference |  | Reference |  |
| 0.50–0.99 | 1.312 (1.024, 1.681) | 0.002 | 1.278 (1.010, 1.617) | <0.05 | 1.273 (1.001, 1.619) | >0.05 |
| 1.00–1.49 | 1.869 (1.302, 2.683) | <0.001 | 1.699 (1.200, 2.407) | 0.003 | 1.686 (1.185, 2.398) | 0.004 |
| ≥1.50 | 2.886 (1.687, 4.938) | <0.001 | 2.531 (1.438, 4.452) | <0.001 | 2.405 (1.393, 4.151) | 0.001 |
| *P* for trend | ＜0.001 | | ＜0.001 | | ＜0.001 | |
|  |  | |  | |  | |
| \| HFHL \| \| \| \| \| \| \| \| --- \| --- \| --- \| --- \| --- \| --- \| --- \| \| <0.50 \| Reference \|  \| Reference \|  \| Reference \|  \| \| 0.50–0.99 \| 1.325 (1.031, 1.702) \| <0.05 \| 1.292 (1.002, 1.667) \| <0.05 \| 1.287 (0.978, 1.700) \| >0.05 \| \| 1.00–1.49 \| 1.825 (1.259, 2.646) \| 0.002 \| 1.660 (1.158, 2.380) \| 0.006 \| 1.643 (1.139, 2.368) \| 0.008 \| \| ≥1.50 \| 2.988 (1.741, 5.127) \| <0.001 \| 2.617 (1.479, 4.631) \| <0.001 \| 2.489 (1.430, 4.331) \| 0.001 \| \| *P* for trend \| ＜0.001 \| \| ＜0.001 \| \| ＜0.001 \| \| \|  \| \| \| \| \| \| \| \| SFHL \| \| \| \| \| \| \| \| <0.50 \| Reference \|  \| Reference \|  \| Reference \|  \| \| 0.50–0.99 \| 1.217 (0.929, 1.595) \| >0.05 \| 1.193 (0.896, 1.588) \| >0.05 \| 1.184 (0.876, 1.601) \| >0.05 \| \| 1.00–1.49 \| 1.304 (0.840, 2.023) \| >0.05 \| 1.258 (0.820, 1.930) \| >0.05 \| 1.192 (0.770, 1.845) \| >0.05 \| \| ≥1.50 \| 2.239 (1.158, 4.332) \| <0.05 \| 2.052 (1.053, 4.000) \| <0.05 \| 2.208 (1.093, 4.460) \| <0.05 \| \| *P* for trend \| ＜0.05 \| \| ＜0.05 \| \| ＜0.05 \| \| \|  \| \| \| \| \| \| \| \| LFHL \| \| \| \| \| \| \| \| <0.50 \| Reference \|  \| Reference \|  \| Reference \|  \| \| 0.50–0.99 \| 1.124 (0.839, 1.506) \| >0.05 \| 1.094 (0.797, 1.501) \| >0.05 \| 1.083 (0.776, 1.510) \| >0.05 \| \| 1.00–1.49 \| 1.048 (0.691, 1.589) \| >0.05 \| 1.011 (0.665, 1.539) \| >0.05 \| 0.958 (0.622, 1.476) \| >0.05 \| \| ≥1.50 \| 2.708 (1.100, 6.667) \| <0.05 \| 2.513 (1.001, 6.309) \| <0.05 \| 2.715 (1.045, 7.055) \| <0.05 \| \| *P* for trend \| >0.05 \| \| >0.05 \| \| >0.05 \| \| \|  \|  \| \|  \| \|  \| \| | | | | | | |
| Tinnitus | | | | | | |
| <0.50 | Reference |  | Reference |  | Reference |  |
| 0.50–0.99 | 1.176 (1.051, 1.316) | <0.05 | 1.143 (0.994, 1.314) | >0.05 | 1.117 (0.951, 1.312) | >0.05 |
| 1.00–1.49 | 1.348 (1.098, 1.655) | 0.005 | 1.295 (1.018, 1.647) | <0.05 | 1.241 (1.005, 1.532) | <0.05 |
| ≥1.50 | 1.557 (1.141, 2.125) | <0.001 | 1.514 (1.109, 2.067) | 0.001 | 1.484 (1.099, 2.004) | 0.003 |
| *P* for trend | ＜0.001 | | ＜0.001 | | ＜0.001 | |

Weighted logistic regression models were adjusted using the same covariate strategy as in Table 2 (Models 1–3), with Model 3 additionally adjusting for tinnitus in the hearing-loss models and for hearing loss in the tinnitus models.

**Supplementary Table S3.** Subgroup analyses of the associations between remnant cholesterol and auditory outcomes

| **Subgroup** | **Positive Proportion** | **OR (95%CI)** | ***P* value** | ***P* for interaction** |
| --- | --- | --- | --- | --- |
| **Hearing loss** | | | | |
| Total | 1421/3169 | 2.434 (1.706, 3.473) | <0.001 |  |
| Age |  |  |  | 0.662 |
| <60 | 386/1724 | 2.339 (1.518, 3.603) | <0.001 |  |
| ≥60 | 1035/1445 | 2.596 (1.327, 5.078) | 0.006 |  |
| Gender |  |  |  | 0.317 |
| Male | 838/1502 | 3.103 (1.794, 5.367) | <0.001 |  |
| Female | 583/1667 | 2.153 (1.283, 3.615) | 0.004 |  |
| BMI |  |  |  | 0.641 |
| Underweight and normal weight | 407/871 | 2.576 (1.118, 5.936) | <0.05 |  |
| Overweight and obesity | 1014/2298 | 2.611 (1.786, 3.816) | <0.001 |  |
| Education level |  |  |  | 0.100 |
| High school graduate or less | 458/836 | 1.374 (0.687, 2.750) | 0.364 |  |
| College or above | 963/2333 | 2.742 (1.879, 4.002) | <0.001 |  |
| Marital status |  |  |  | 0.109 |
| Cohabiting | 914/2057 | 3.319 (2.084, 5.286) | <0.001 |  |
| Alone | 507/1112 | 1.738 (1.057, 2.903) | <0.05 |  |
| Family PIR |  |  |  | 0.131 |
| <1.3 | 416/863 | 1.742 (1.033, 3.047) | <0.05 |  |
| 1.3-3.5 | 597/1189 | 2.470 (1.396, 4.370) | 0.002 |  |
| ≥3.5 | 408/1117 | 3.260 (1.786, 5.950) | <0.001 |  |
| Smoking |  |  |  | 0.801 |
| Never smoker | 639/1664 | 2.389 (1.313, 4.347) | 0.005 |  |
| Former smoker | 547/953 | 2.779 (1.661, 4.650) | <0.001 |  |
| Current smoker | 235/552 | 2.388 (1.342, 4.248) | 0.004 |  |
| Drinking |  |  |  | 0.256 |
| Non-drinker | 337/728 | 1.288 (0.639, 2.594) | 0.474 |  |
| Moderate drinker | 699/1400 | 3.239 (1.867, 5.621) | <0.001 |  |
| Heavy drinker | 385/1041 | 2.444 (1.340, 4.457) | 0.004 |  |
| Noise exposure |  |  |  | <0.001 |
| No | 706/1725 | 1.354 (0.862, 2.128) | 0.186 |  |
| Yes | 715/1444 | 4.208 (2.371, 7.469) | <0.001 |  |
| Hypertension |  |  |  | 0.300 |
| No | 663/1767 | 2.848 (1.647, 4.924) | <0.001 |  |
| Yes | 758/1402 | 2.212 (1.349, 3.628) | 0.002 |  |
| Diabetes |  |  |  | 0.954 |
| No | 1105/2592 | 2.447 (1.621, 3.693) | <0.001 |  |
| Yes | 316/577 | 2.692 (1.189, 6.097) | <0.05 |  |
| CVD |  |  |  | 0.475 |
| No | 1147/2768 | 2.338 (1.553, 3.521) | <0.001 |  |
| Yes | 274/401 | 9.554 (2.773, 32.914) | <0.001 |  |
|  | | | | |
| **Tinnitus** | | | | |
| Total | 1102/4922 | 1.642 (1.311, 2.056) | <0.001 |  |
| Age |  |  |  | 0.308 |
| <60 | 499/2657 | 1.392 (1.032, 1.877) | <0.05 |  |
| ≥60 | 603/2265 | 2.397 (1.630, 3.524) | <0.001 |  |
| Gender |  |  |  | 0.246 |
| Male | 473/2157 | 1.895 (1.364, 2.633) | <0.001 |  |
| Female | 629/2765 | 1.499 (1.057, 2.125) | <0.05 |  |
| BMI |  |  |  | 0.747 |
| Underweight and normal weight | 327/1419 | 1.931 (1.187, 3.141) | 0.009 |  |
| Overweight and obesity | 775/3503 | 1.718 (1.312, 2.251) | <0.001 |  |
| Education level |  |  |  | 0.994 |
| High school graduate or less | 375/1512 | 1.698 (1.094, 2.635) | <0.05 |  |
| College or above | 727/3410 | 1.672 (1.305, 2.143) | <0.001 |  |
| Marital status |  |  |  | 0.443 |
| Cohabiting | 683/3112 | 1.689 (1.288, 2.215) | <0.001 |  |
| Alone | 419/1810 | 1.668 (1.102, 2.524) | <0.05 |  |
| Family PIR |  |  |  | 0.479 |
| <1.3 | 344/1371 | 1.732 (1.010, 2.968) | <0.05 |  |
| 1.3-3.5 | 436/1850 | 1.511 (1.004, 2.273) | <0.05 |  |
| ≥3.5 | 322/1701 | 1.782 (1.131, 2.808) | <0.05 |  |
| Smoking |  |  |  | 0.407 |
| Never smoker | 488/2546 | 1.815 (1.288, 2.559) | <0.001 |  |
| Former smoker | 390/1500 | 1.505 (1.007, 2.251) | <0.05 |  |
| Current smoker | 224/876 | 1.899 (1.107, 3.260) | <0.05 |  |
| Drinking |  |  |  | 0.972 |
| Non-drinker | 265/1185 | 1.874 (0.972, 3.614) | 0.061 |  |
| Moderate drinker | 495/2179 | 1.683 (1.167, 2.428) | 0.006 |  |
| Heavy drinker | 342/1558 | 1.702 (1.215, 2.387) | 0.003 |  |
| Noise exposure |  |  |  | <0.05 |
| No | 663/3171 | 1.275 (0.940, 1.730) | 0.116 |  |
| Yes | 439/1751 | 2.313 (1.612, 3.320) | <0.001 |  |
| Hypertension |  |  |  | 0.099 |
| No | 585/2874 | 1.437 (1.067, 1.935) | <0.05 |  |
| Yes | 517/2048 | 2.033 (1.448, 2.855) | <0.001 |  |
| Diabetes |  |  |  | 0.271 |
| No | 930/4205 | 1.606 (1.271, 2.030) | <0.001 |  |
| Yes | 172/717 | 2.277 (1.143, 4.538) | <0.05 |  |
| CVD |  |  |  | 0.422 |
| No | 903/4251 | 1.624 (1.265, 2.085) | <0.001 |  |
| Yes | 199/671 | 2.352 (1.318, 4.197) | 0.005 |  |

Weighted logistic regression models were based on Model 3 (adjusted for demographic, socioeconomic, lifestyle, and clinical covariates). ORs (95% CIs) were estimated within each subgroup to examine potential effect modification. *P*-for-interaction values were derived from cross-product terms between remnant cholesterol and each subgroup variable in the fully adjusted model.

**Supplementary Figure S1.** Interactive effects of remnant cholesterol and noise exposure on auditory outcomes

Restricted cubic spline models showing the interactive effects of RC and noise exposure on (A) hearing loss and (B) tinnitus. Weighted logistic regression models were adjusted as in Model 3. Solid lines represent adjusted odds ratios, and shaded areas indicate 95% confidence intervals, illustrating stronger dose–response relationships among participants exposed to noise.


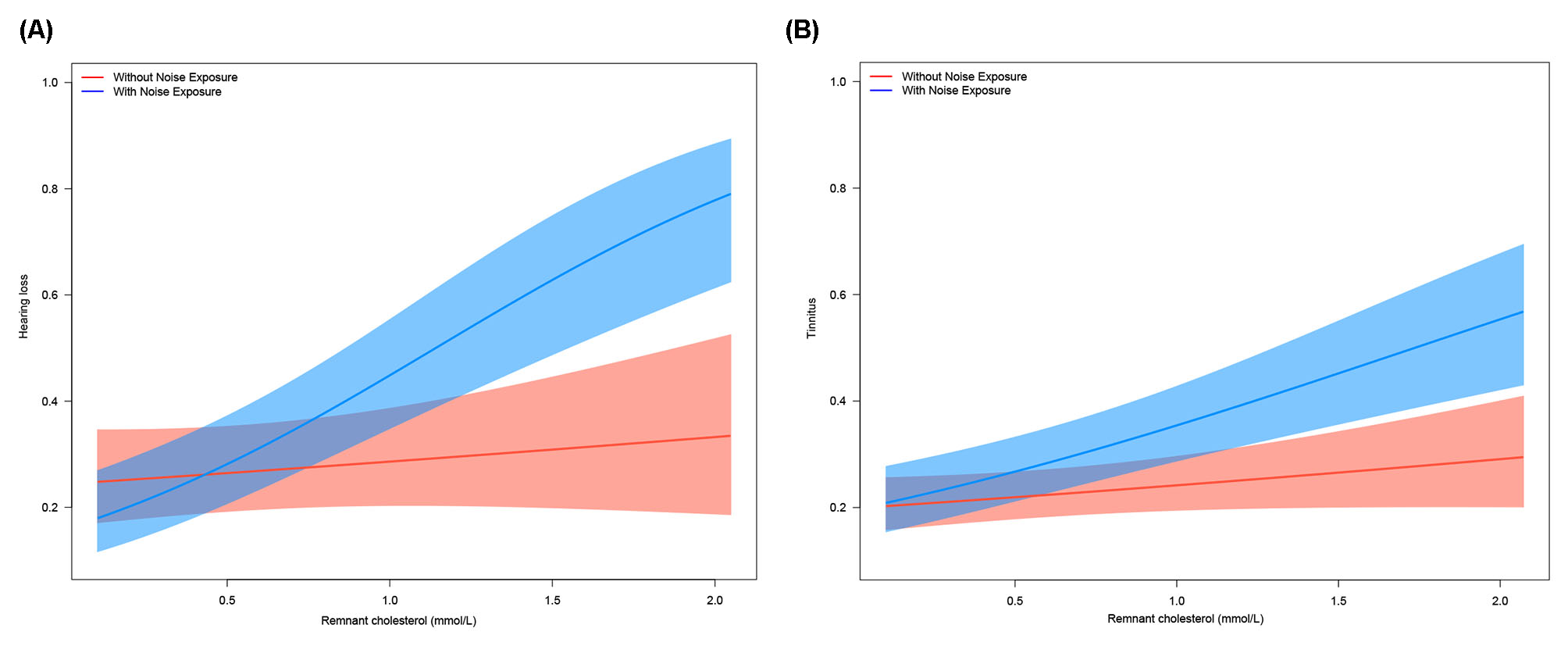

Supplement: Supplementary file 2 — Supplementary Material 2. [file 12944_2026_2910_MOESM2_ESM.docx]
